# Identification and validation of a KRAS-macrophage-associated gene signature as prognostic biomarkers and potential therapeutic targets in melanoma

**DOI:** 10.3389/fimmu.2025.1566432

**Published:** 2025-06-18

**Authors:** Beichen Cai, Qian Lin, Ruonan Ke, Jiaqi Yu, Lu Chen, Xuejun Ni, Hekun Liu, Xiuying Shan, Biao Wang

**Affiliations:** ^1^ Department of Plastic Surgery, the First Affiliated Hospital of Fujian Medical University, Fuzhou, China; ^2^ Department of Plastic Surgery, National Regional Medical Center, Binhai Campus of the First Affiliated Hospital, Fujian Medical University, Fuzhou, China; ^3^ Fujian Key Laboratory of Translational Research in Cancer and Neurodegenerative Diseases, Institute for Translational Medicine, School of Basic Medical Sciences, Fujian Medical University, Fuzhou, China

**Keywords:** melanoma, KRAS signaling, macrophage infiltration, prognostic biomarker, immune microenvironment, single-cell RNA sequencing

## Abstract

**Introduction:**

Skin cutaneous melanoma (SKCM) is a highly aggressive form of cancer with poor prognosis, characterized by significant molecular and immune heterogeneity. The activation of KRAS signaling pathways is implicated in melanoma progression, yet its role in shaping the tumor microenvironment, particularly in macrophage infiltration, remains poorly understood.

**Methods:**

A comprehensive multi-platform approach was employed, analyzing gene expression data from the Gene Expression Omnibus (GEO) and The Cancer Genome Atlas (TCGA) databases. Gene set enrichment analysis (GSEA) was utilized to characterize the molecular pathways associated with KRAS signaling. Single-cell RNA sequencing (scRNA-seq) was leveraged to investigate the cellular heterogeneity within the SKCM tumor microenvironment, and macrophage populations were categorized using the Monocle2 algorithm. A KRAS-Macrophage Prognostic Associated Gene (KMPAG) signature was developed by integrating these findings, followed by validation using a least absolute shrinkage and selection operator (LASSO) regression model. The prognostic value of the KMPAG signature was assessed through its correlation with clinical outcomes, immune cell infiltration patterns, response to therapy, drug sensitivity, and miRNA-gene regulatory interactions. Cell-cell communication within the SKCM microenvironment was explored using the “CellChat” tool. Experimental validation of gene expression was performed via immunohistochemistry (IHC) and functional assays in gene-modified melanoma cell lines.

**Results:**

Twenty-two genes involved in KRAS signaling were identified as critical for patient survival. Single-cell analysis revealed nine distinct cell populations within the SKCM microenvironment, leading to the construction of the KMPAG risk model, which incorporated three key genes—CLEC4A, CXCL10, and LAT2. This signature effectively reclassified macrophage subsets, offering improved diagnostic and prognostic capabilities. Furthermore, the KMPAG signature correlated with a range of clinical parameters, including immune infiltration levels, tumor stage, and therapy response. The model also provided insights into the immune landscape of SKCM, facilitating the prediction of responses to immunotherapy. Functional assays demonstrated that downregulation of CLEC4A significantly promoted melanoma cell proliferation, migration, and invasion.

**Conclusion:**

This study highlights the importance of KRAS signaling and macrophage infiltration in melanoma prognosis. The KMPAG gene signature presents a novel prognostic tool, offering insights into personalized treatment strategies and predictive biomarkers for immunotherapy in SKCM. Further exploration of CLEC4A’s role in melanoma progression may provide new therapeutic avenues for targeted intervention.

## Highlights

- Identification of Genes Associated with KRAS Pathway Activity: A set of 22 genes linked to KRAS signaling was identified, correlating with patient survival outcomes in melanoma.
- Single-Cell Profiling: Comprehensive single-cell RNA sequencing revealed nine principal cell types within the melanoma tumor microenvironment, enhancing the understanding of cellular heterogeneity.
- KMPAG Risk Model Development: A prognostic risk model, the KRAS-Macrophage Prognostic Associated Gene (KMPAG) signature, was developed using CLEC4A, CXCL10, and LAT2, offering significant potential for clinical applications.
- Immune Landscape Reclassification: The KMPAG model successfully reclassified macrophage subsets in SKCM, improving diagnostic accuracy and providing new insights into immune microenvironment dynamics.
- Functional Role of CLEC4A: Downregulation of CLEC4A significantly enhanced melanoma cell proliferation, migration, and invasion, suggesting its potential as a therapeutic target.

## 1 Introduction

Cutaneous melanoma stands as one of the most aggressive forms of skin cancer, characterized by its pronounced metastatic propensity and high mortality rate (1, 2). Despite notable advancements in diagnostic imaging, surgical techniques, and systemic treatments, melanoma continues to pose a formidable challenge in clinical oncology (3). Traditional treatment paradigms have predominantly centered on surgical resection and cytotoxic chemotherapy, yet the outcomes remain suboptimal in patients exhibiting advanced or recurrent disease (4). Recent strides in immunotherapy and targeted agents, including immune checkpoint inhibitors and inhibitors targeting driver gene mutations, have expanded therapeutic options and prolonged survival for some individuals with advanced melanoma (5, 6). However, intrinsic and acquired resistance mechanisms persist, underscoring the necessity for more robust predictive biomarkers, refined prognostic stratification tools, and innovative therapeutic strategies (7).

Melanoma development involves a multifaceted interplay between oncogenic mutations and a highly dynamic tumor microenvironment (8). Genomic analyses have revealed that oncogenic mutations in genes such as BRAF and NRAS dominate the landscape of cutaneous melanoma, frequently initiating aberrant signaling pathways that foster unchecked cellular proliferation and survival (9–11). Nevertheless, a subgroup of melanomas appears to exhibit activation of the KRAS pathway or dysregulation of key components therein (12, 13). While KRAS mutations in cutaneous melanoma occur less frequently than BRAF or NRAS mutations, KRAS-linked signaling has garnered attention in other malignancies and exhibits the potential to influence melanoma cell proliferation and phenotypic plasticity (14). The precise mechanistic underpinnings of KRAS pathway interactions with other known driver mutations in cutaneous melanoma remain insufficiently elucidated, warranting a deeper investigation into whether KRAS-associated gene dysregulation confers survival disadvantages or heightened metastatic capacity (15).

Alongside these genomic alterations, the tumor microenvironment exerts profound impacts on disease progression and patient prognosis (16). Complex cellular and cytokine networks, supported by a heterogeneous population of immune cells, fibroblasts, endothelial cells, and other stromal components, collectively orchestrate an evolving niche that modulates tumor growth and therapy responses (17). Of particular relevance, macrophages constitute a substantial immune cell subset within the tumor microenvironment and display a spectrum of functional states, broadly classified into M1-like and M2-like phenotypes (18). While M1-like macrophages predominantly elicit antitumor activity, M2-like macrophages generally support tumor expansion and immune evasion, thereby correlating with worse clinical outcomes (19). Investigating macrophage-associated signaling in conjunction with oncogenic pathways holds promise for enhancing prognostic accuracy and unveiling points of therapeutic intervention (20).

Owing to the intricate tumor–immune interplay in melanoma, the exploration of single-cell transcriptomics has emerged as a powerful approach to delineate the heterogeneity underlying melanoma lesions and the immune cells that infiltrate them (21). Unlike bulk transcriptomics, single-cell RNA sequencing allows precise profiling of distinct cellular populations, revealing critical insights into cell subtype composition, functional states, and developmental trajectories (22). This high-resolution perspective illuminates complex intercellular interactions and has the potential to highlight novel biomarkers, define disease progression states, and identify determinants of therapeutic response (23).

The present investigation sought to integrate diverse transcriptomic datasets, including single-cell RNA sequencing and bulk gene expression profiling, to elucidate the role of KRAS-related signaling in melanoma and examine how macrophage phenotypes contribute to clinical outcomes (24). Particular emphasis was placed on identifying survival-related hub genes within the KRAS pathway, scrutinizing the distribution and functional polarization of macrophages, and constructing a prognostic gene signature that underscores the interplay between KRAS signaling and macrophage infiltration (25). A comprehensive study design incorporated multiple public melanoma cohorts, rigorous bioinformatic analyses, and subsequent experimental validation in cell-based models (26). By uncovering the molecular features that correlate with tumor aggressiveness and immune dysregulation, the analysis aspired to forge a deeper understanding of melanoma pathogenesis and facilitate the development of more personalized therapies (27).

To translate computational findings into clinically actionable insights, a specialized prognostic model was built, termed the KRAS-Macrophage Prognostic Associated Gene (KMPAG) signature (28). This model integrated survival-related genes linked to KRAS pathway activation and robustly validated their significance across independent patient cohorts (29). The proposed KMPAG signature underscores the importance of three genes—CLEC4A, CXCL10, and LAT2—in modulating tumor–immune crosstalk and predicting patient outcomes (30). Beyond prognostic utility, each candidate biomarker may hold therapeutic implications, including their capacity to predict responses to immunotherapy or targeted pharmacologic agents (31).

Moreover, immune infiltration patterns, estimated through computational deconvolution algorithms, provided further support for the linkage between a KRAS-associated gene signature and macrophage-driven immune responses (32). In order to solidify the mechanistic foundation, functional assays were performed to assess whether modulating the expression of these hub genes influences melanoma cellular behavior (33). The resulting functional data shed light on the potential of CLEC4A, in particular, to modulate proliferative, migratory, and invasive phenotypes (34). Hence, this multipronged approach, spanning statistical modeling, single-cell analytics, and targeted functional assays, offers a nuanced framework for understanding melanoma biology (35).

## 2 Materials and methods

### 2.1 Data acquisition and processing

A comprehensive dataset integration strategy was employed to obtain transcriptomic profiles from both bulk and single-cell sequencing platforms. Publicly accessible repositories served as the primary sources of melanoma-related gene expression data (36), encompassing multiple Gene Expression Omnibus (GEO) datasets, specifically GSE72056 (previously described (21)), which included single-cell RNA sequencing (scRNA-seq) data from 4,645 cells across 19 melanoma patients, and bulk microarray datasets GSE8401, GSE15605, GSE46517, and GSE65904 (214 melanoma specimens with prognostic annotations) (37). Information regarding BRAF mutation status was not uniformly available or annotated across all utilized GEO datasets. Additionally, RNA-seq profiles from 477 SKCM specimens were obtained from The Cancer Genome Atlas (TCGA) repository (26), for which corresponding somatic mutation data, including BRAF status, was available and utilized in correlative analyses where specified, and 812 normal skin tissue samples were sourced from the Genotype-Tissue Expression (GTEx) database as controls for comparative analyses (38). Each dataset underwent stringent curation to exclude incomplete clinical or survival information, followed by standardized pre-processing steps including log2-based transformation, background correction, and normalization.

To facilitate cross-cohort comparisons, we implemented a rigorous batch effect correction protocol. First, we assessed the presence and magnitude of batch effects using principal component analysis (PCA) and hierarchical clustering of samples before correction. Distinct clustering patterns by data source were observed, confirming the presence of technical variation. For correction, we employed the ComBat algorithm from the “sva” R package (version 3.42.0) with default parameters, using data source (TCGA, GEO datasets, GTEx) as the batch variable while preserving biological variation associated with sample type (tumor vs. normal) and clinical parameters. The effectiveness of batch correction was validated through post-correction PCA visualization, which demonstrated successful integration of samples across data sources while maintaining biological separation between tumor and normal samples. Additionally, we confirmed that gene-gene correlation structures were preserved after correction by comparing correlation matrices pre- and post-adjustment (Pearson correlation between matrices = 0.92). For single-cell RNA sequencing data, we implemented the mutual nearest neighbors (MNN) batch correction method via the “batchelor” R package (version 1.10.0) to address patient-specific effects while preserving cell-type heterogeneity, with correction effectiveness verified by UMAP visualization. All analyses involving cross-dataset comparisons were performed using these batch-corrected expression matrices.

### 2.2 Gene set enrichment analysis

Gene Set Enrichment Analysis was conducted to link observed gene expression patterns to underlying biological processes and pathways associated with melanoma progression. Utilizing the “clusterProfiler” package in R (39), genes were ranked based on differential expression metrics derived from comparisons between high-risk and low-risk patient cohorts identified through the prognostic risk model. Pre-defined gene sets, including those related to KRAS signaling and other oncogenic pathways, were employed to evaluate the enrichment of specific molecular signatures (40). Enrichment scores were calculated by traversing the ranked gene list and aggregating contributions from the gene sets, thereby identifying overrepresented pathways at the extremes of the ranking. Single-sample Gene Set Enrichment Analysis (ssGSEA) was additionally performed using the Gene Set Variation Analysis (GSVA) package in R (41) to quantify the relative abundance of 28 immune cell phenotypes within the tumor microenvironment, utilizing bespoke gene signatures for each immune cell type.

### 2.3 Quality control and single-cell gene expression data analysis

Single-cell transcriptomic data from the GSE72056 dataset were subjected to rigorous quality control to ensure high-fidelity interpretation of cellular states and gene expression patterns. Cells with fewer than 200 or more than 10,000 detected genes, as well as those exhibiting more than 20% mitochondrial gene expression, were excluded to remove low-quality or doublet cells. Following filtering, data normalization was performed using the “NormalizeData” function in the Seurat package (42), and highly variable genes were identified using the “FindVariableFeatures” function (method = vst, nfeatures = 2000) to capture transcriptionally diverse features. Principal component analysis was conducted, and the optimal number of principal components (17) was determined using “ElbowPlot” and “JackStrawPlot” functions. Clustering was achieved via the “FindClusters” function (resolution = 1.5), resulting in 4,611 high-quality cells for analysis. Dimensionality reduction was performed using t-distributed Stochastic Neighbor Embedding (t-SNE) through the “RunTSNE” function, facilitating the visualization of distinct cell populations. Cluster-specific marker genes were identified using the “FindAllMarkers” function, and cell type annotations were assigned using the “SingleR” (Single-cell Recognition) package (43) based on established human cellular markers, thereby generating a detailed atlas of the tumor microenvironment, including key immune populations such as macrophages and T lymphocytes.

For single-cell RNA sequencing analysis, initial clustering was performed using the Seurat package with multiple resolution parameters tested (0.4, 0.6, 0.8, 1.0, 1.2, 1.5), with the final resolution of 1.5 selected based on silhouette width analysis and biological interpretation of resulting clusters. Cluster marker genes were identified using the Wilcoxon Rank Sum test with a minimum log fold-change threshold of 0.25 and adjusted p-value < 0.05. For subclustering of macrophage populations, an optimal resolution of 0.5 was determined through iterative testing and evaluation of biological coherence using established macrophage marker panels. To functionally annotate the identified macrophage subpopulations from scRNA-seq data, M1 and M2 signature scores were calculated for each macrophage cell using the ‘AddModuleScore’ function in Seurat, based on established M1 (CD80, CD86, IL1B, TNF, IRF5) and M2 (CD163, CD206/MRC1, IL10, ARG1, CCL18) gene sets.

### 2.4 Single-cell trajectory analysis

Pseudotime trajectory analysis was performed on selected single-cell populations, with a focus on macrophage subtypes, to elucidate lineage relationships and differentiation states within the melanoma microenvironment. The Monocle2 framework was utilized, employing the default DDR-Tree (Discriminative Dimensionality Reduction via Learning Tree) algorithm parameters (44). Marker genes identified from Seurat clustering were selected for their relevance to macrophage polarization and functional states. These genes informed the low-dimensional embedding, capturing the transcriptional continuum from pro-tumoral to anti-tumoral macrophage phenotypes. Branch Expression Analysis Modeling (BEAM) was applied to identify genes driving branch-specific differentiation, highlighting critical junctures in cellular state transitions. Visualization of pseudotime progressions and branch points was achieved through color-coded plots, which illustrated the dynamic evolution of macrophage functionality in relation to tumor progression.

### 2.5 Establishment of the KRAS-macrophage prognostic associated gene signature

To elucidate the prognostic significance of the KRAS pathway in skin cutaneous melanoma (SKCM), univariate Cox proportional hazards regression analyses were conducted on both TCGA-SKCM and GSE65904 datasets to identify genes within the KRAS signaling cascade significantly associated with overall survival. Genes overlapping between these datasets and intersecting with macrophage marker genes from the GSE72056 single-cell dataset were selected as candidate hub genes. The Least Absolute Shrinkage and Selection Operator (LASSO) regression method (45) was then applied to these candidates within the TCGA-SKCM cohort to construct a robust prognostic model, mitigating overfitting by penalizing the absolute size of regression coefficients. The optimal penalty parameter (λ) was determined via ten-fold cross-validation based on the minimum mean cross-validated error criterion using the cv.glmnet function. The performance during this internal cross-validation within the TCGA training cohort yielded a mean Area Under the ROC Curve (AUC) of ^[insert mean AUC value,e.g.,0.72 ± 0.05]^, indicating reasonable robustness in the model selection process. The risk score for each patient was calculated as the sum of the expression levels of the selected genes multiplied by their corresponding LASSO-derived coefficients. Patients were stratified into high- and low-risk groups based on the median risk score, and Kaplan–Meier survival analysis (46) was performed to compare overall survival between these groups. The prognostic accuracy of the KMPAG signature was validated in an external cohort (GSE65904) using similar stratification and survival analysis methods, thereby confirming the model’s predictive robustness and clinical relevance in independent melanoma populations.

For the LASSO regression model, the optimal penalty parameter (λ) was determined through 10-fold cross-validation using the “cv.glmnet” function. The value of λ that minimized the cross-validation error (λ.min = 0.0382) was selected for the final model. For robustness, we also evaluated the model using λ.1se (λ = 0.0817, the largest value of λ such that the error was within one standard error of the minimum), which yielded similar gene selection. The final risk score calculation incorporated coefficients derived from λ.min to maximize predictive accuracy.

### 2.6 Functional enrichment analysis of risk model genes

Functional enrichment analyses were conducted to characterize the biological implications of the genes comprising the KMPAG risk model, focusing on pathways and processes implicated in tumor progression and immune regulation. Differentially expressed genes (DEGs) between high-risk and low-risk groups were identified using stringent criteria (adjusted p-value < 0.05 and |Log2 fold-change| ≥ 1). These DEGs were subjected to Gene Ontology (GO) and Kyoto Encyclopedia of Genes and Genomes (KEGG) pathway enrichment analyses using the “clusterProfiler” package (39). GO analysis categorized gene functions into Biological Process, Cellular Component, and Molecular Function, elucidating the functional attributes of the risk-associated genes. KEGG pathway analysis identified significant metabolic and signaling pathways enriched in each risk subgroup, highlighting immune-related processes and oncogenic signaling cascades (47). Multiple hypothesis testing was controlled using false discovery rate (FDR) adjustments to ensure statistical robustness.

### 2.7 Evaluation of immune cell infiltration and tumor microenvironment composition

A comprehensive assessment of the immunological landscape was conducted to elucidate the relationship between the prognostic gene model and distinct immune cell populations within each tumor sample from The Cancer Genome Atlas – Skin Cutaneous Melanoma (TCGA-SKCM) and GSE65904 cohorts. Utilizing the CIBERSORT (Cell type Identification By Estimating Relative Subsets of RNA Transcripts) algorithm in conjunction with the LM22 (Leukocyte signature matrix containing 22 immune cell types) signature matrix (32), bulk transcriptomic data were computationally deconvoluted to estimate the relative abundances of 22 immune cell subsets per patient. Pre-processing steps included normalization and log-transformation to ensure consistency across diverse gene expression platforms. Samples exhibiting low confidence or outlier metrics, as determined by CIBERSORT’s inference quality parameters, were either cautiously examined or excluded from subsequent analyses. The proportions of key immune cell types, including CD8^+^ and CD4^+^ T cells, B cells, natural killer cells, dendritic cells, and macrophage subtypes (M0, M1, M2), were quantified within both high-risk and low-risk patient groups. Comparative analyses of immune infiltration patterns were performed using non-parametric statistical tests with p-values adjusted for multiple comparisons to identify significant differences.

### 2.8 Assessment of immunotherapeutic responsiveness

The Tumor Immune Dysfunction and Exclusion (TIDE) computational framework was employed to evaluate the potential for immune evasion within gene expression profiles derived from tumor specimens of TCGA-SKCM and GSE65904 cohorts (48). TIDE integrates two primary mechanisms of immunotherapy resistance: T-cell dysfunction within the tumor microenvironment and T-cell exclusion by the tumor stroma. By analyzing these aspects, TIDE generates composite scores that serve as quantitative metrics for predicting patient responses to immune checkpoint blockade (ICB) therapies. Lower TIDE scores are indicative of a higher likelihood of favorable responses to immunotherapy, whereas elevated scores correlate with a reduced probability of achieving therapeutic benefits from ICB. Additionally, ancillary scores such as microsatellite instability (MSI) and activities of protumoral macrophage subsets were considered to provide a comprehensive assessment of immunological barriers. To externally validate the KMPAG signature’s ability to predict response to immune checkpoint blockade (ICB), we obtained transcriptomic data and clinical response information from a published cohort of metastatic melanoma patients treated with anti-PD1 therapy (GSE78220). KMPAG risk scores were calculated for each patient in the GSE78220 dataset using the formula derived from the TCGA training set. Patients were stratified into high- and low-risk groups based on the median risk score. The association between KMPAG risk groups and clinical response (Complete Response/Partial Response vs. Stable Disease/Progressive Disease) was assessed using Fisher’s exact test. Kaplan-Meier analysis and log-rank tests were used to compare progression-free survival (PFS) between the predicted risk groups.

### 2.9 Correlation between KMPAG signature and drug sensitivity

Drug sensitivity analyses were conducted to determine whether the KRAS-Macrophage Prognostic Associated Gene (KMPAG) signature could predict differential responsiveness to chemotherapeutic and targeted therapeutic agents in TCGA-SKCM and GSE65904 cohorts. Utilizing the “pRRophetic” R package (49), the half-maximal inhibitory concentration (IC_50_) values for a range of approved and investigational drugs were estimated based on individual patient gene expression profiles. Patients were stratified into high-risk and low-risk groups according to their KMPAG-derived risk scores, and differential drug sensitivities between these groups were assessed using boxplot visualizations of the predicted IC_50_ values. Statistical analyses employed rigorous thresholds, including adjusted p-values, to identify significant differences in drug responsiveness. Particular focus was placed on agents currently used in the treatment of advanced melanoma, such as alkylating agents, BRAF and MEK inhibitors, and immunomodulatory drugs.

### 2.10 Construction of the miRNA–mRNA regulatory network

To elucidate the post-transcriptional regulatory mechanisms influencing the KRAS-Macrophage Prognostic Associated Gene (KMPAG) signature, a comprehensive microRNA (miRNA)–messenger RNA (mRNA) regulatory network was constructed. Potential microRNAs targeting the KMPAG genes (CLEC4A, CXCL10, LAT2) were identified by querying four established bioinformatics databases: miRDB, miRWalk, RNA22, and TargetScan (50–53). Only miRNAs predicted to target these genes by at least two databases were selected to ensure robustness of predictions. The intersections of miRNA predictions were visualized using upset plots to highlight shared miRNA candidates. Subsequently, Cytoscape software was employed to create an interactive miRNA–mRNA network, illustrating the regulatory relationships between the identified miRNAs and their target genes (54).

### 2.11 Analysis of cell-cell communication modalities

Intercellular communication dynamics within the melanoma tumor microenvironment were comprehensively analyzed using the CellChat algorithm (version 1.1.0) (55). This analytical framework integrates a curated repository of biologically relevant ligands, receptors, cofactors, and cytokines to infer potential ligand-receptor interactions between different cell populations identified through single-cell RNA sequencing data from the GSE72056 dataset. Pre-processed single-cell expression matrices were imported into CellChat, where parameters such as gene detection thresholds and communication probability models were meticulously optimized to enhance the accuracy of inferred interactions. The CellChat algorithm systematically identified and quantified communication events, constructing interaction networks that depict the strength and prevalence of ligand-receptor pairs between cell types. Visualization of these networks was achieved through chord diagrams, heatmaps, and circos plots generated by the CellChat Explorer web interface, facilitating the interpretation of complex communication patterns. Special emphasis was placed on immunologically significant pathways, including the CXCL chemokine signaling axis, which plays a pivotal role in immune cell recruitment and modulation within the tumor microenvironment.

For cell-cell communication analysis using CellChat, we employed stringent filtering criteria to ensure biological relevance of identified interactions. An interaction probability threshold of 0.05 was applied, meaning that interactions with probabilities below this threshold were excluded from the analysis. Communication patterns were identified using a permutation test with 1,000 permutations, and only interactions with p < 0.05 were considered significant. For visualization of interaction networks, we applied a minimum edge weight filter of 0.25 to highlight the strongest interactions while maintaining network interpretability.

### 2.12 Immunohistochemistry staining

Immunohistochemical staining was performed on formalin-fixed, paraffin-embedded benign nevi and melanoma tissue sections to validate the protein expression levels of candidate genes identified through bioinformatics analyses. Tissue sections, 4–5 μm thick, were deparaffinized in xylene and rehydrated through a graded series of ethanol solutions. Antigen retrieval was achieved using a citrate buffer (pH 6.0) or EDTA buffer (pH 8.0), followed by quenching of endogenous peroxidase activity with 3% hydrogen peroxide. Non-specific binding was blocked using a serum-based blocking solution appropriate for the primary antibody host species. Sections were subsequently incubated overnight at 4°C with primary antibodies targeting proteins of interest, at concentrations optimized through preliminary pilot experiments. After extensive washing, sections were treated with Horseradish Peroxidase (HRP)-conjugated secondary antibodies, followed by visualization with a 3,3’-diaminobenzidine (DAB) substrate-chromogen system to detect protein localization. Slides were counterstained with hematoxylin to highlight cellular morphology, dehydrated, cleared, and mounted for microscopic examination. Immunoreactivity was assessed by experienced pathologists using semi-quantitative scoring systems that evaluated both staining intensity and the percentage of positive cells (56).

### 2.13 Cell culture and transfection

Human melanoma cell lines, including A375 (known to harbor the BRAF V600E mutation), SK-MEL-2, A2058, and MV3, were procured from the American Type Culture Collection (ATCC; Manassas, VA, USA) (57) and cultured under standardized conditions to ensure consistency and reliability of experimental results. Cells were maintained in high-glucose Dulbecco’s Modified Eagle Medium (DMEM) supplemented with 10% fetal bovine serum (FBS) and 1% penicillin/streptomycin solution, and incubated at 37°C in a humidified atmosphere containing 5% CO_2_, adhering to stringent protocols for cell culture. Transfection experiments aimed at modulating CLEC4A expression were conducted using viral vectors engineered and packaged by Hanbio Biotechnology (Shanghai, China). Specifically, the A375 cell line was employed for establishing stable melanoma cell lines with either knockdown or overexpression of CLEC4A. The shRNA and overexpression constructs utilized were pHBLV-U6-MCS-CMV-ZsGreen-PGK-PURO and pHBLV-CMV-MCS-EF1-Zsgreen-T2A-PURO, respectively. The short hairpin RNA (shRNA) sequence designed for CLEC4A silencing was sh-GCAAGAAGAATCTGCTTATTT, while the overexpression sequence was meticulously crafted to optimize gene expression. Transfection was performed when cells reached approximately 40% confluence, utilizing the synthesized viral solutions in combination with the transfection enhancer RNAFit to enhance efficiency. Following a 72-hour post-transfection period, the integration efficiency of the viral vectors was assessed via fluorescence microscopy, ensuring the successful incorporation of the genetic material. Subsequently, puromycin selection was initiated to isolate stably transfected clones, thereby enabling the establishment of reliable cell models for downstream functional assays (58).

### 2.14 Real-time quantitative polymerase chain reaction and western blot assay

To validate the alterations in gene expression induced by transfection, both RT-qPCR and Western blot assays were meticulously performed. Total RNA was extracted from cultured melanoma cells using the Trizol reagent, followed by DNase I treatment to eliminate genomic DNA contaminants. Two micrograms of the purified RNA were reverse transcribed using the PrimeScript RT Master Mix, and the resulting cDNA was subjected to quantitative PCR using the SYBR Green Real-time PCR Master Mix kit under specified thermal cycling conditions. Relative mRNA expression levels were quantified using the 2^−ΔΔCt method (59), with primers designed for Homo sapiens CLEC4A and ACTB serving as the internal control. Similarly, for the analysis of clinical tissue samples, total RNA was extracted from 8 benign nevi and 12 melanoma tissues using Trizol reagent, followed by cDNA synthesis and RT-qPCR performed as described above to compare their relative expression.

For protein analysis, cellular lysates were prepared using Radioimmunoprecipitation assay (RIPA) buffer supplemented with a protease inhibitor cocktail and 1 mM Phenylmethylsulfonyl fluoride (PMSF), followed by centrifugation at 12,000 g for 10 minutes at 4°C to obtain the protein extract. Equal amounts of protein were resolved via Sodium dodecyl-sulfate polyacrylamide gel electrophoresis (SDS-PAGE) and transferred to polyvinylidene difluoride (PVDF) membranes. Membranes were blocked with 5% non-fat milk and incubated overnight with primary antibodies against CLEC4A and beta-actin (ACTB), followed by probing with horseradish peroxidase-conjugated secondary antibodies and visualization using the Enhanced Chemiluminescence (ECL) detection system. Band intensities were quantified using appropriate imaging software, normalized to beta-actin levels, and analyzed to confirm the efficacy of gene modulation at both the transcriptional and translational levels (60). For Western blot validation in clinical tissues, protein extracts were obtained from 6 benign nevi and 6 melanoma tissues using RIPA buffer. Subsequent procedures for SDS-PAGE, membrane transfer, incubation with the aforementioned primary antibodies for CLEC4A and ACTB, and ECL detection were performed identically to those for cellular lysates, with these experiments conducted in duplicate.

### 2.15 Cell proliferation assays

Cellular proliferation was assessed through a combination of the kFluor555 Click-iT EdU Imaging Assay, colony formation assays, and the Cell Counting Kit-8 (CCK-8) assay, providing comprehensive insights into the proliferative dynamics of melanoma cells upon gene modulation. In the EdU incorporation assay, cells were incubated with 5-ethynyl-2’-deoxyuridine (EdU) for two hours to label newly synthesized DNA, followed by fixation, permeabilization, and the Click-iT reaction under light-protected conditions. Nuclei were counterstained with Hoechst 33342 for fluorescent imaging. Concurrently, colony formation assays were performed by seeding 2 × 10³ cells per well in 6-well plates and allowing colonies to form over a two-week period. Colonies were then fixed, stained with crystal violet, and enumerated. Additionally, the CCK-8 assay was used to measure metabolic activity as a surrogate for cell viability by quantifying optical density (OD) at 450 nm (61).

### 2.16 Cell migration and invasion assays

The migratory and invasive capabilities of melanoma cells were evaluated using the wound healing (scratch) assay and the Transwell invasion assay. For the wound healing assay, cells were seeded in six-well plates and grown to near confluence, followed by the creation of a consistent linear wound using a sterile pipette tip under serum-starved conditions. Micrographs of the wound area were captured at 0 and 24 hours, and wound closure was quantified using ImageJ software (62). In parallel, the invasive potential of the cells was assessed using Transwell inserts coated with Matrigel. After 24 hours of incubation, non-invaded cells were removed, and invaded cells were fixed, stained, and quantified microscopically (63).

### 2.17 Statistical analyses

Comprehensive statistical analyses were performed to interpret the experimental data, employing a suite of robust methodologies to identify significant differences and underlying trends. Comparative analyses between two independent groups utilized the Student’s t-test for normally distributed data or the Wilcoxon rank-sum test for non-parametric data. For comparisons involving multiple groups, one-way analysis of variance (ANOVA) was employed, followed by appropriate *post hoc* tests. Categorical variables were analyzed using the chi-square test or Fisher’s exact test as appropriate. Survival data were evaluated using Kaplan-Meier survival curves and the log-rank test, while univariate and multivariate Cox proportional hazards regression models were applied to compute hazard ratios (HR) and 95% confidence intervals (CI). Correlation analyses between gene expression levels and immune cell fractions were conducted using Pearson’s or Spearman’s correlation coefficients (64). In high-dimensional expression datasets, multiple testing corrections were implemented to mitigate Type I errors. Quantitative data were presented as mean ± standard deviation (SD) from at least three independent biological replicates. All statistical computations were executed using R (v4.2.2) (65) and GraphPad Prism (v9.0) (66), leveraging specialized packages including “Seurat” for single-cell RNA-seq data analysis, “ggplot2” for advanced graphical representations, “limma” for differential expression analysis, “clusterProfiler” for functional enrichment analyses, “survival” and “survminer” for survival analyses, and “glmnet” for fitting generalized linear models with penalized maximum likelihood. A significance threshold of p<0.05 was established *a priori*, with *, **, and *** denoting p<0.05, p<0.01, and p<0.001, respectively.

## 3 Results

### 3.1 KRAS pathway enrichment and identification of survival-related hub genes in melanoma

**Figure 1:**
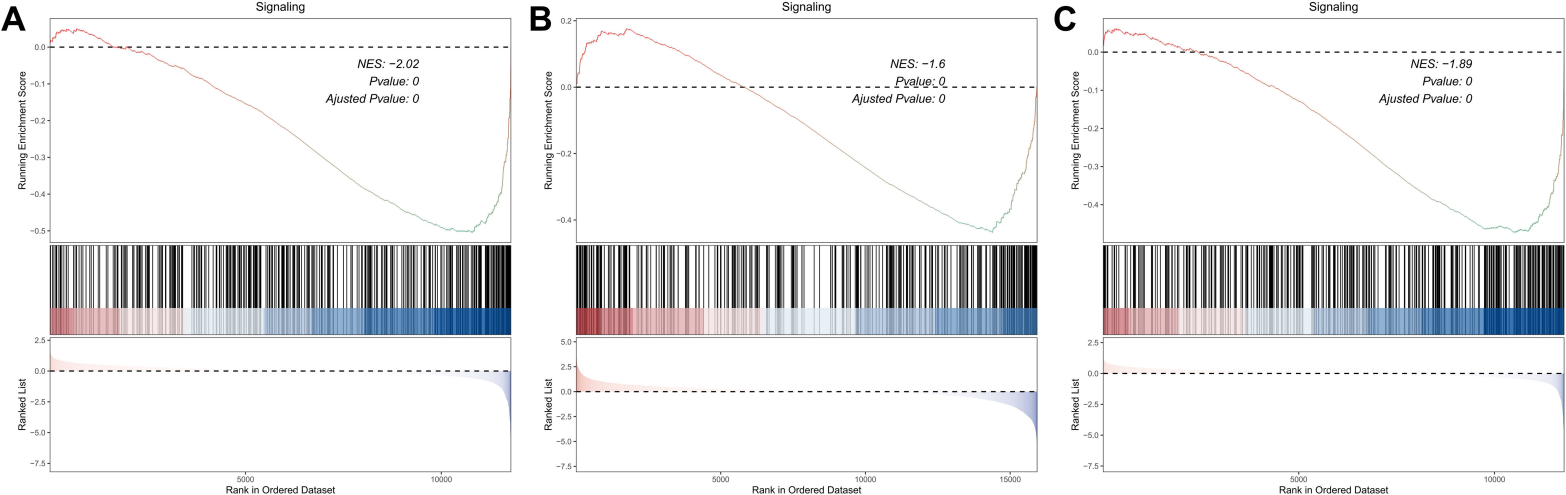
Investigation of the KRAS Signaling Pathway Enrichment in SKCM Datasets via GSEA. **(A)** GSEA comparing primary melanomas vs. normal skin (GSE15605). **(B, C)** GSEA contrasting metastatic vs. primary melanomas (GSE8401, GSE46517), showing increased KRAS pathway activation in advanced disease.

**Figure 2:**
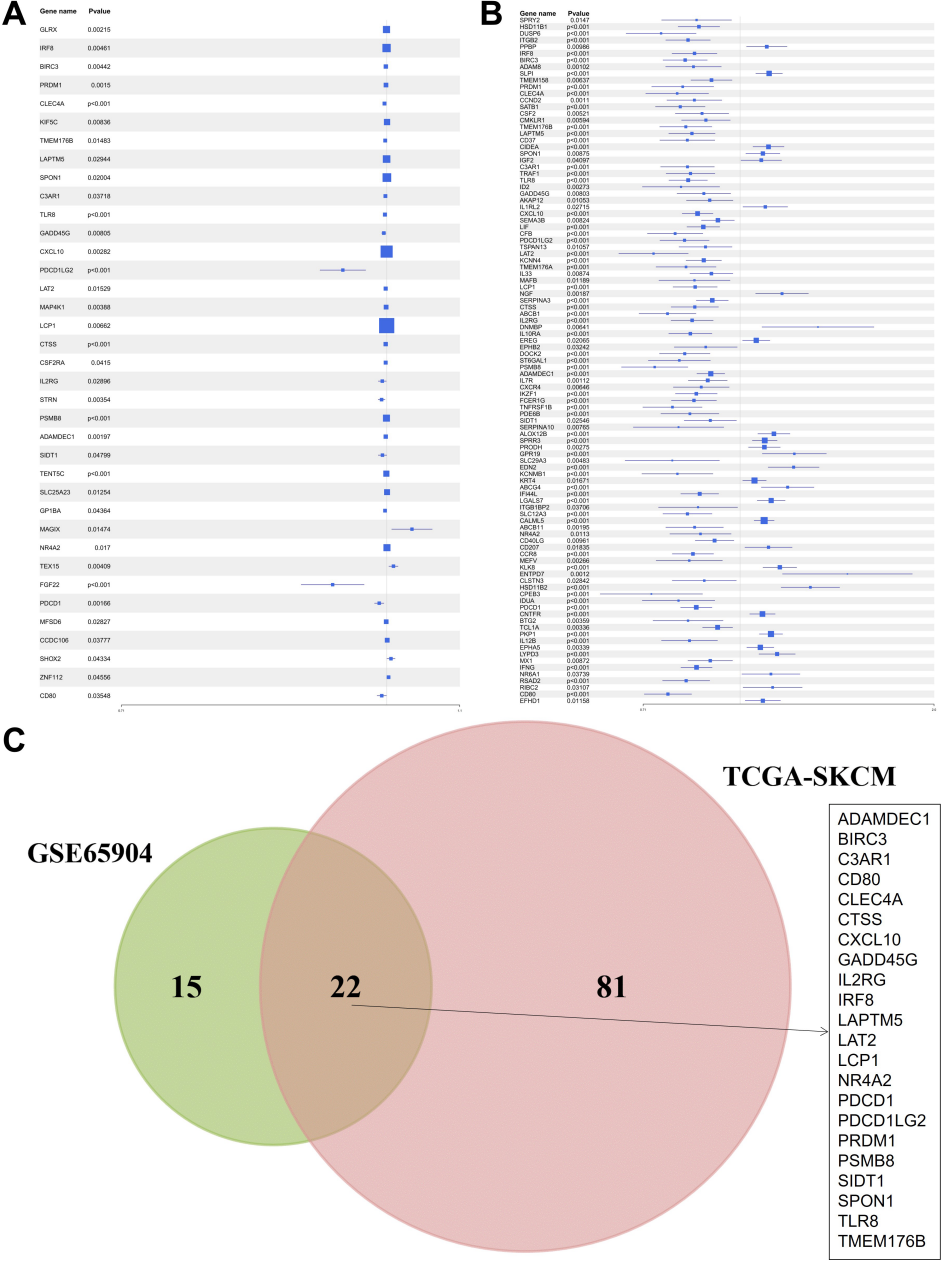
Identification of hub genes related to survival within the KRAS signaling pathway. **(A)** Univariate Cox regression results for KRAS pathway genes in GSE65904. **(B)** Parallel analysis in TCGA-SKCM. **(C)** Venn diagram showing the 22 common survival-associated hub genes.

An integrative analysis was conducted to investigate the role of KRAS-associated signaling pathways in the pathogenesis and clinical outcomes of skin cutaneous melanoma (SKCM). Gene Set Enrichment Analysis (GSEA) was performed on multiple transcriptomic datasets, including GSE15605, GSE8401, and GSE46517, comparing melanoma tissues to normal skin and primary tumors to metastatic lesions (**Figures 1A–C**). The analysis revealed a significant enrichment of KRAS signaling pathways, indicating the potential involvement of molecular mechanisms associated with KRAS pathway activity in melanoma initiation and progression. Subsequently, a Univariate Cox Proportional Hazards Regression Model was employed to identify survival-associated genes within the KRAS signaling pathway across two independent datasets: GSE65904 and TCGA-SKCM. This approach identified 37 survival-related hub genes from the GSE65904 dataset and 103 from the TCGA-SKCM dataset (**Figures 2A, B**). The intersection of these gene sets yielded 22 hub genes that are intricately linked to both KRAS signaling activity and patient survival (**Figure 2C**). These hub genes encompass diverse functional categories, including regulators of cytokine signaling, immune response modulators, and key players in cell cycle control. The identification of these genes underscores their potential pivotal roles in SKCM pathogenesis and their utility as prognostic biomarkers and targets for therapeutic interventions.

### 3.2 Single-cell profiling reveals tumor microenvironment complexity and macrophage infiltration

**Figure 3:**
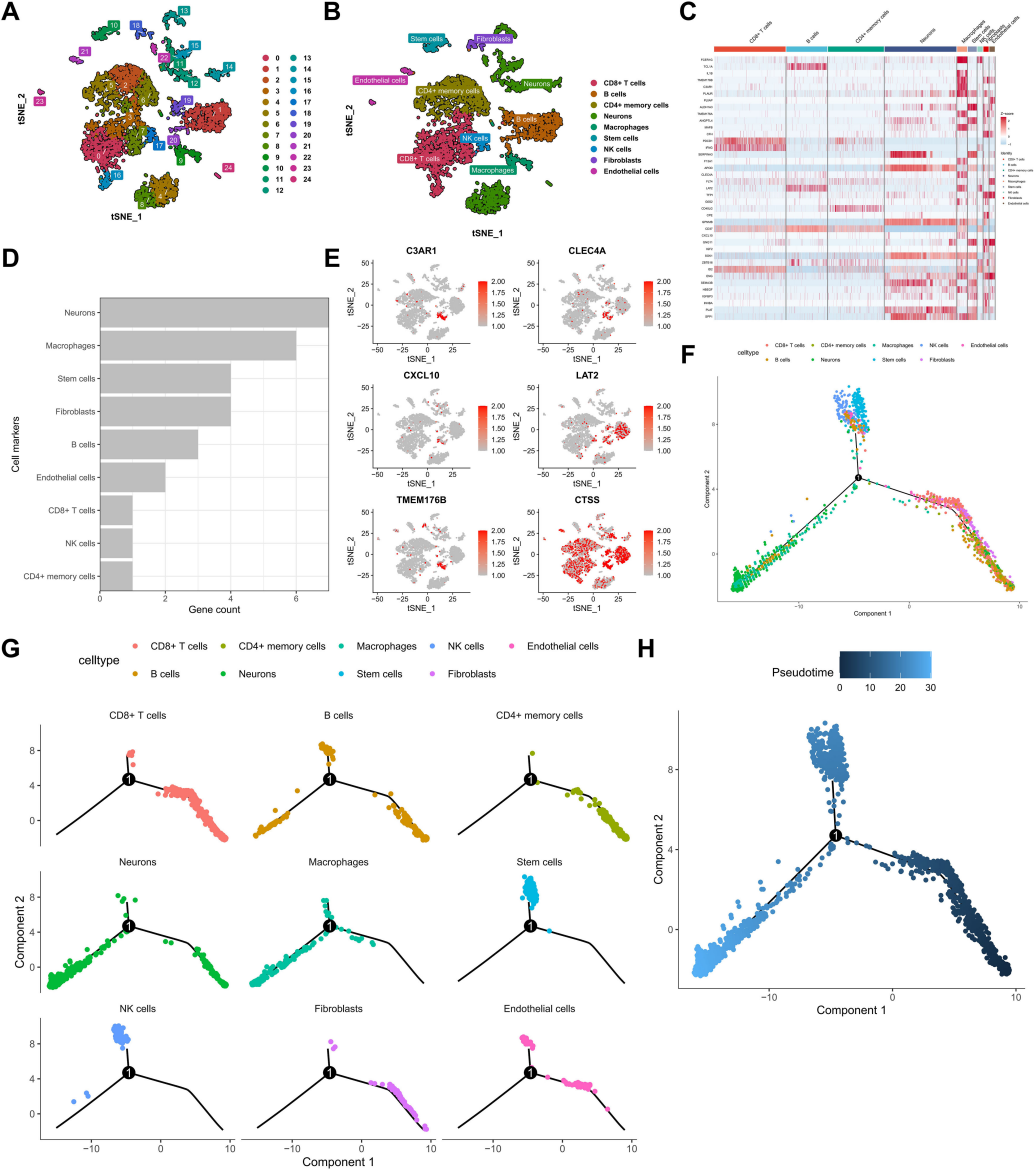
Single-cell RNA sequencing reveals cellular heterogeneity and KRAS hub gene expression patterns. **(A)** t-SNE clustering of melanoma single cells. **(B)** Annotation of principal cell types. **(C)** Heatmap of KRAS pathway marker expression across clusters. **(D, E)** Intersection of KRAS hub genes with cell-type markers, highlighting macrophages. **(F, G)** Monocle2 pseudotime trajectory analysis across all identified cell populations.

Following the identification of 22 KRAS pathway-associated hub genes significantly correlated with survival in bulk SKCM transcriptomes (**Figure 2**), we sought to dissect the cellular heterogeneity of the tumor microenvironment (TME) to understand which cell types contribute to this prognostic signal, particularly focusing on immune populations known to interact with oncogenic signaling. To achieve this, we leveraged single-cell RNA sequencing (scRNA-seq) data from the GSE72056 dataset (**Figure 3A**). After rigorous quality-control filtering, clustering analyses using t-distributed stochastic neighbor embedding (t-SNE) identified 24 distinct cell clusters, which were subsequently annotated into nine major cell types: CD8^+^ T cells, B cells, CD4^+^ T memory cells, neurons, macrophages, stem cells, Natural Killer (NK) cells, fibroblasts, and endothelial cells (**Figure 3B**, **Supplementary Figure S1**). Each cell type was characterized by the expression of canonical marker genes, with a comprehensive heatmap illustrating the expression of KRAS-related and featured genes across these clusters (**Figure 3C**). Intersection analysis revealed that among the 22 hub genes, seven intersected with marker genes in the neurons cluster, six in macrophages, four each in stem cells and fibroblasts, three in B cells, two in endothelial cells, and one each in CD8^+^ T cells, NK cells, and CD4^+^ T memory cells (**Figure 3D**). Macrophages exhibited the highest number of intersecting genes, prompting a detailed localization of these six genes within the macrophage population (**Figure 3E**). Pseudotime trajectory analysis using the Monocle2 algorithm demonstrated that neurons and macrophages followed similar developmental trajectories, primarily situated in the early pseudotime states, whereas immune cells such as CD8^+^ T cells, CD4^+^ memory cells, and B cells were predominantly positioned in later states (**Figures 3F, G**). Furthermore, single-sample Gene Set Enrichment Analysis (ssGSEA) was utilized to score TCGA-SKCM samples based on the expression of cell subgroup markers. Subsequently, the TCGA-SKCM samples were bifurcated into two distinct groups predicated on the optimal cutoff value of each cell score, followed by a comparative analysis of the survival disparities between the groups, thus crafting the survival curve. Kaplan-Meier survival analysis revealed that higher infiltration levels of macrophages, B cells, CD8^+^ T cells, and stem cells were associated with improved overall survival (OS), whereas increased levels of fibroblasts and endothelial cells correlated with poorer prognosis (**Supplementary Figure S2**). These findings highlight the complex cellular landscape of SKCM and underscore the significant prognostic impact of macrophage infiltration within the tumor microenvironment.

### 3.3 Development and validation of a three-gene prognostic signature linked to KRAS and macrophage infiltration

**Figure 4:**
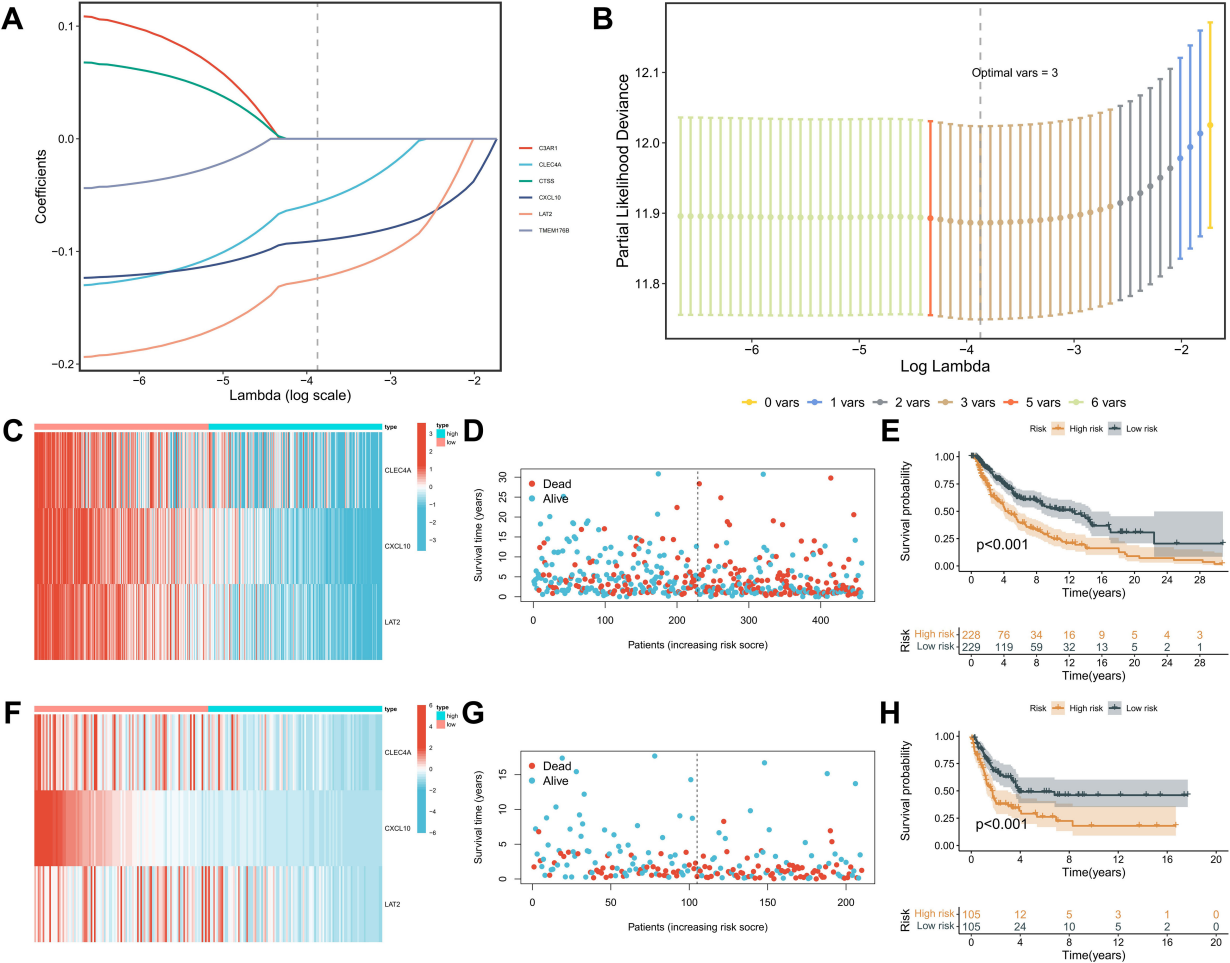
Construction and validation of the KMPAG prognostic risk model. **(A, B)** LASSO regression analysis selecting CLEC4A, CXCL10, and LAT2. **(C–E)** Risk score distribution, survival status, and Kaplan-Meier OS curves for high/low-risk groups in the TCGA training set. **(F-H)** Validation in the GSE65904 cohort.

Building upon the identification of KRAS-related and macrophage-associated hub genes, a prognostic signature was developed using the Least Absolute Shrinkage and Selection Operator (LASSO) regression method implemented via the “glmnet” R package. From the initial six macrophage-relevant genes, LASSO regression selected three genes—CLEC4A, CXCL10, and LAT2—constituting the KRAS-Macrophage Prognostic Associated Gene (KMPAG) signature (**Figures 4A, B**). The risk score was calculated using the formula: risk score = −0.0564 × CLEC4A + −0.0906 × CXCL10 + −0.1239 × LAT2. SKCM patients from the TCGA dataset were stratified into high-risk and low-risk groups based on the median risk score (**Figures 4C, D**). Kaplan-Meier survival analysis demonstrated that the high-risk group had significantly poorer OS compared to the low-risk group (P < 0.001) (**Figure 4E**). The prognostic robustness of the KMPAG signature was validated in an independent cohort from the GSE65904 dataset, where high-risk patients also exhibited significantly reduced OS (P < 0.01) (**Figures 4F–H**). Further analysis revealed that CLEC4A and CXCL10 were predominantly expressed in macrophages, while LAT2 was expressed in macrophages, B cells, and NK cells (**Supplementary Figure S3A, B**; **Supplementary Figure S4**). These results validate the KMPAG signature as a reliable prognostic tool and highlight CLEC4A, CXCL10, and LAT2 as potential biomarkers for personalized therapeutic strategies in SKCM.

### 3.4 Identification of macrophage subpopulations and their functional implications in melanoma progression

**Figure 5:**
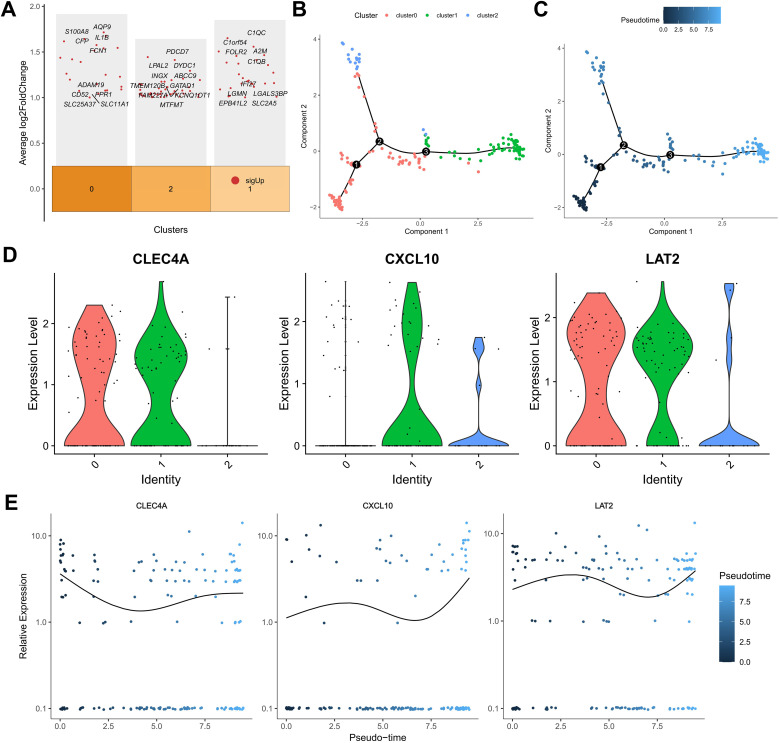
Single-cell analysis of macrophage subpopulations. **(A)** Heatmap of differentially expressed genes in macrophage subclusters. **(B)** Pseudotime trajectory reconstruction of macrophage state transitions. **(C)** Branch plots indicating potential differentiation points. **(D, E)** Expression trends of KMPAG genes (CLEC4A, CXCL10, LAT2) along the macrophage pseudotime trajectory.

A comprehensive re-analysis of the single-cell clustering results was conducted to elucidate the heterogeneity within the macrophage compartment, given the significant contribution of macrophage-associated genes to the prognostic signature. Utilizing the clustertree depicted (**Supplementary Figure S5A**), an optimal resolution threshold of 0.5 was determined, enabling the segregation of the macrophage population into three distinct subclusters. Differential expression analysis across these subclusters identified a set of genes exhibiting significant variance, including those involved in immunostimulation, antigen processing, and immunosuppression (**Figure 5A**). Subsequent Gene Ontology (GO) and Kyoto Encyclopedia of Genes and Genomes (KEGG) pathway analyses revealed that cluster 0 was enriched for genes related to RAGE receptor binding, immune receptor activity, inhibitory Major Histocompatibility Complex (MHC) class I receptor activity, and actin binding, indicating an immunoregulatory phenotype. Cluster 1 displayed significant enrichment in MHC class II protein complex binding, oxidoreductase activity specific to NAD(P)H and quinone compounds, and active transmembrane transporter activity driven by oxidoreduction processes, suggesting a role in oxidative metabolism and antigen presentation. In contrast, cluster 2 was predominantly associated with ribosomal structural components, receptor antagonist activity, and signaling receptor inhibitor activity (**Supplementary Figures S5B, C**). KEGG analysis further highlighted that cluster 0 was involved in pathways such as Shigellosis, C-type lectin receptor signaling, TNF signaling, Salmonella infection, and NF-κB signaling, while cluster 1 was linked to Lysosome, Phagosome, Intestinal immune network for IgA production, Rheumatoid arthritis, and glycan degradation pathways. Cluster 2 was primarily associated with Ribosome and Coronavirus disease – COVID-19 pathways, underscoring its potential involvement in protein synthesis and viral response mechanisms.

To assess their functional polarization, we calculated M1 and M2 signature scores. cluster 1 exhibited significantly higher M1 scores, aligning with pro-inflammatory functions and antigen presentation pathways (e.g., MHC class II complex binding) identified in GO/KEGG analysis. Conversely, cluster 0 showed relatively higher M2 scores and enrichment for immunoregulatory pathways (e.g., RAGE receptor binding, C-type lectin receptor signaling), potentially representing TAMs with immunosuppressive or tissue-remodeling roles, although canonical M2 markers like CD163 were not among the top DEGs for this cluster. cluster 2, enriched for ribosomal components, displayed lower M1/M2 scores, possibly representing a less polarized or distinct functional state. While these computational clusters provide insights, their precise correspondence to canonical M1/M2 phenotypes or established TAM subtypes (e.g., lipid-associated macrophages) requires further validation. Pseudotime trajectory analysis using the Monocle2 algorithm revealed a developmental continuum within the macrophage subpopulations (**Figures 5B, C**). Cells from cluster 0 were predominantly positioned at the early pseudotime states, progressing towards clusters 2 and 1 over time, indicative of differentiation pathways influenced by the tumor microenvironment. Expression dynamics of the KMPAG-associated genes demonstrated that CLEC4A was highly expressed in clusters 0 and 1, CXCL10 was predominantly expressed in clusters 1 and 2, and LAT2 was consistently expressed across all clusters (**Figure 5D**). The temporal evolution of these genes along the pseudotime axis suggested their roles in macrophage differentiation and functional modulation. These findings substantiate the diverse functional states of macrophages in SKCM and their interaction with KRAS-related oncogenic signals, highlighting the importance of macrophage subset composition in influencing tumor progression and patient prognosis.

### 3.5 High diagnostic accuracy of the KRAS-macrophage prognostic signature for melanoma

**Figure 6:**
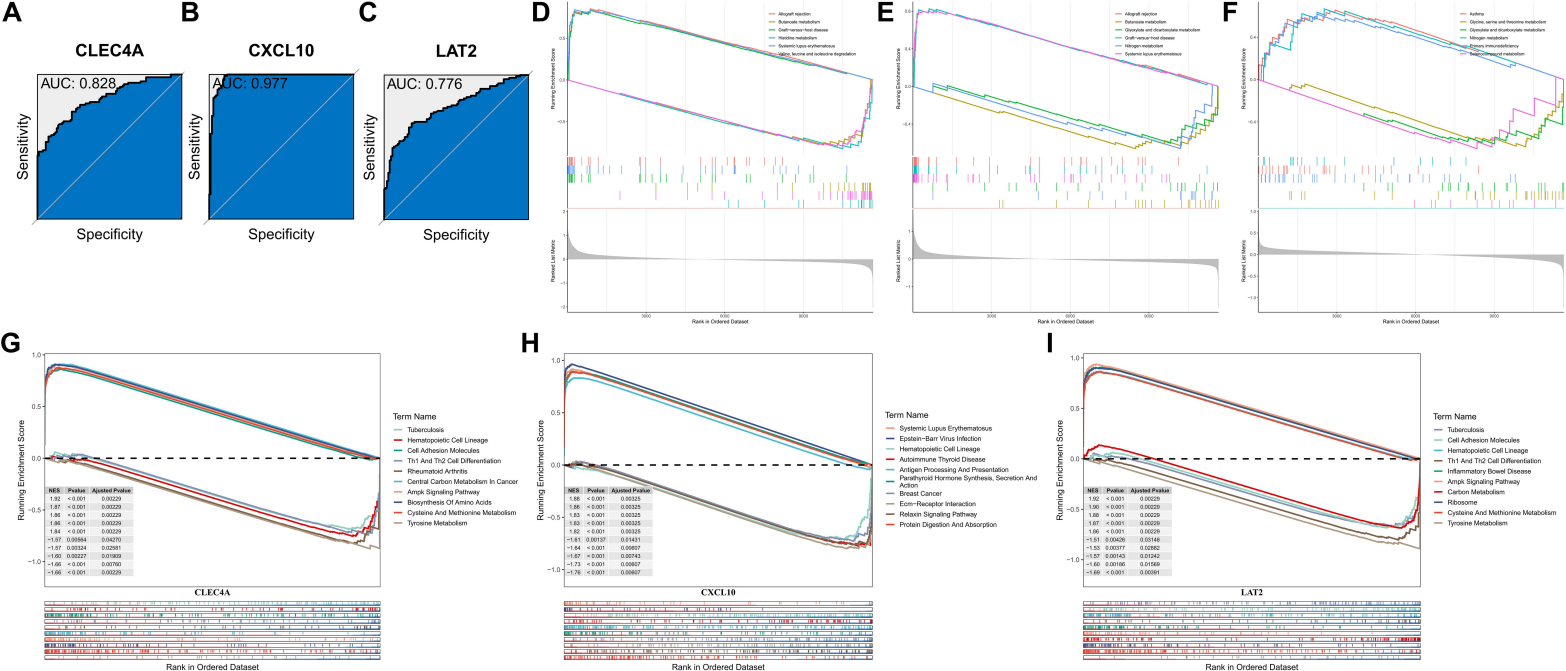
Diagnostic accuracy and functional enrichment analysis of KMPAG hub genes. **(A–C)** ROC curves evaluating the diagnostic performance of CLEC4A, CXCL10, and LAT2 using TCGA-SKCM vs. GTEx data. **(D–F)** GSEA plots for representative pathways associated with each hub gene. **(G–I)** GSEA plots for representative disease-related pathways associated with each hub gene.

The diagnostic potential of the KMPAG signature was rigorously evaluated using Receiver Operating Characteristic (ROC) curve analyses based on data from the TCGA-SKCM cohort. Each signature gene—CLEC4A, CXCL10, and LAT2—demonstrated substantial diagnostic accuracy, with Area Under the Curve (AUC) values of 0.828, 0.977, and 0.776, respectively (**Figures 6A–C**). These results indicate that the KMPAG genes possess strong discriminatory power in distinguishing SKCM tissues from normal controls and stratifying patients based on risk profiles. Furthermore, pathway interaction analyses revealed that CLEC4A was significantly associated with Allograft Rejection, Butanoate Metabolism, Graft-versus-host Disease, Histidine Metabolism, Systemic Lupus Erythematosus, and Valine, Leucine, and Isoleucine Degradation pathways (**Figure 6D**). CXCL10 was linked to Allograft Rejection, Butanoate Metabolism, Glyoxylate and Dicarboxylate Metabolism, Graft-versus-host Disease, Nitrogen Metabolism, and Systemic Lupus Erythematosus pathways (**Figure 6E**), while LAT2 was associated with Asthma, Glycine, Serine and Threonine Metabolism, Glyoxylate and Dicarboxylate Metabolism, Nitrogen Metabolism, Primary Immunodeficiency, and Selenocompound Metabolism pathways (**Figure 6F**).

Single-gene GSEA further elucidated the involvement of KMPAG genes in various biological processes and disease pathways. CLEC4A was implicated in Tuberculosis, Hematopoietic Cell Lineage, Cell Adhesion Molecules, Th1 and Th2 Cell Differentiation, Rheumatoid Arthritis, Central Carbon Metabolism in Cancer, AMPK Signaling Pathway, Biosynthesis of Amino Acids, Cysteine and Methionine Metabolism, and Tyrosine Metabolism (**Figure 6G**). CXCL10 was associated with Systemic Lupus Erythematosus, Epstein-Barr Virus Infection, Hematopoietic Cell Lineage, Autoimmune Thyroid Disease, Antigen Processing and Presentation, Parathyroid Hormone Synthesis, Secretion and Action, Breast Cancer, ECM-Receptor Interaction, Relaxin Signaling Pathway, and Protein Digestion and Absorption (**Figure 6H**). LAT2 was linked to Tuberculosis, Cell Adhesion Molecules, Hematopoietic Cell Lineage, Th1 and Th2 Cell Differentiation, Inflammatory Bowel Disease, AMPK Signaling Pathway, Carbon Metabolism, Ribosome, Cysteine and Methionine Metabolism, and Tyrosine Metabolism (**Figure 6I**). These comprehensive analyses underscore the diagnostic and prognostic relevance of the KMPAG signature and highlight their integral roles in key biological processes underlying SKCM pathogenesis.

### 3.6 Clinical relevance of the KMPAG signature correlates with disease severity and prognosis

**Figure 7:**
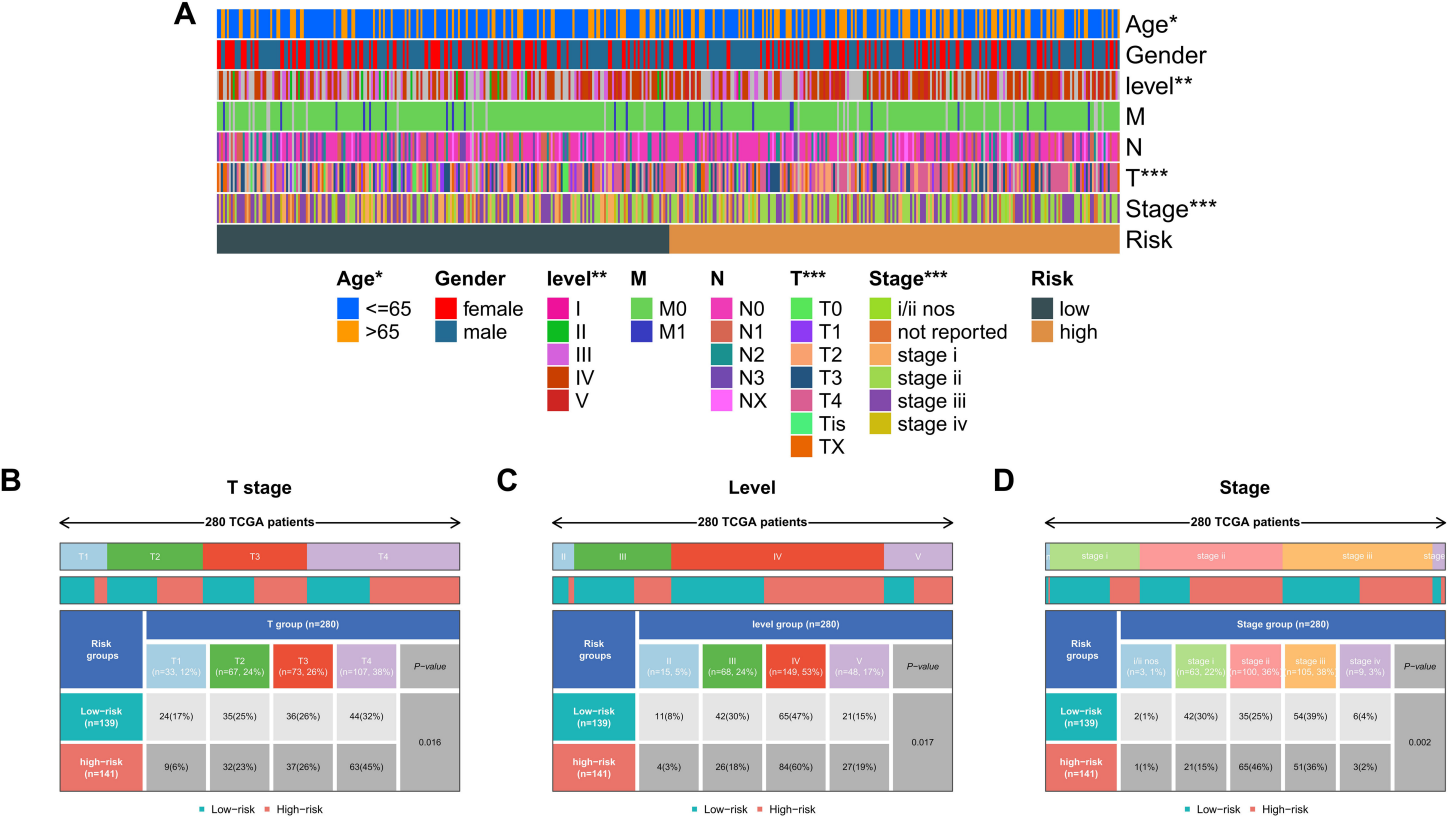
Clinical correlations of the KMPAG signature in SKCM. **(A)** Heatmap associating KMPAG risk scores with clinical factors (T stage, ulceration, etc.). **(B–D)** Boxplots and statistical tests linking risk scores to T category, Clark level, and pathological stage in TCGA-SKCM.

To validate the clinical relevance of the KMPAG signature, an extensive correlation analysis was performed between the risk scores derived from the signature and various clinicopathological parameters within the TCGA-SKCM dataset (**Figure 7A**). Patients were stratified into high-risk and low-risk groups based on the median risk score. Comparative analysis revealed that the low-risk group had a higher proportion of T1 stage patients (17%) and a lower proportion of T4 stage patients (32%) compared to the high-risk group, which had 6% T1 and 45% T4 patients (p = 0.016, chi-square test) (**Figure 7B**). Similarly, the low-risk group exhibited a greater number of patients in lower levels (II and III), whereas the high-risk group predominantly comprised level IV patients (47% vs. 60%, p = 0.017, chi-square test) (**Figure 7C**). Furthermore, analysis of disease staging indicated that the low-risk cohort contained more stage III patients (39%), while the high-risk cohort had a higher representation of stage II patients (46%, p = 0.002, chi-square test) (**Figure 7D**). Multivariable Cox proportional hazards models, adjusted for confounding variables such as age and sex, affirmed the independent prognostic value of the KMPAG signature (**Figure 7E**). These findings demonstrate that the KMPAG signature is significantly correlated with key clinical parameters, reinforcing its potential utility as a prognostic biomarker for stratifying SKCM patients based on disease severity and predicting clinical outcomes.

### 3.7 Immune landscape distinctions between high- and low-risk patient cohorts based on KMPAG signature

**Figure 8:**
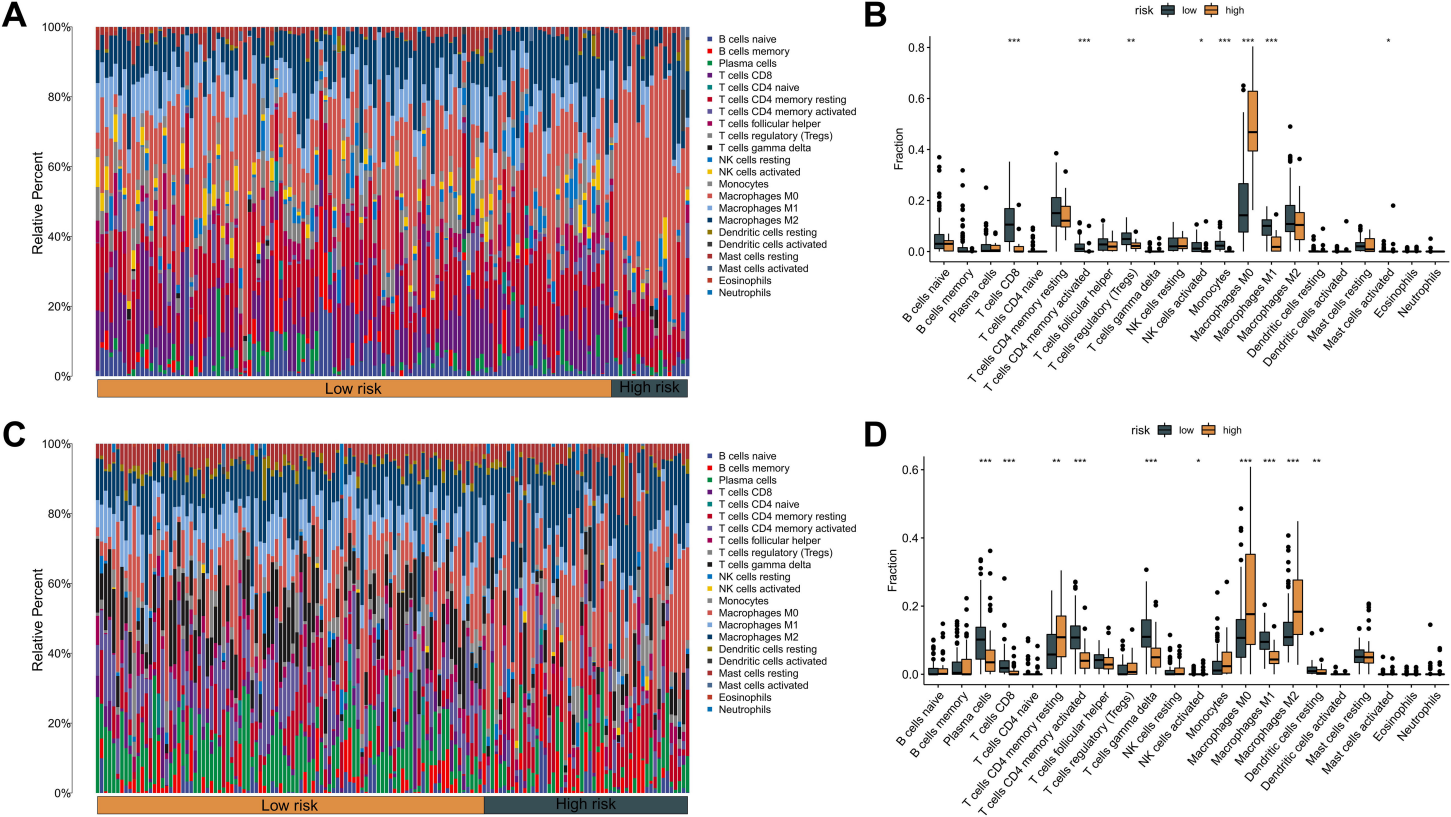
Association between KMPAG signature and tumor immune infiltration. **(A, B)** CIBERSORT estimation and comparison of 22 immune cell types in high- vs. low-risk groups within the TCGA-SKCM cohort. **(C, D)** Corresponding immune infiltration analysis in the GSE65904 cohort. *p < 0.05; **p < 0.01; ***p < 0.001.

An extensive immunological profiling of tumor samples was conducted to determine whether the KMPAG risk classification corresponded to distinct immune cell infiltration states. Utilizing the CIBERSORT (Cell type Identification By Estimating Relative Subsets of RNA Transcripts) algorithm alongside the LM22 signature matrix, the proportions of 22 distinct immune cell types were quantified in each SKCM specimen within the TCGA dataset (**Figure 8A**). Comparative analysis between high-risk and low-risk cohorts revealed that high-risk samples exhibited a significant enrichment of non-activated macrophages (M0) and a marked reduction in the infiltration of CD8^+^ T cells, activated CD4^+^ memory T cells, regulatory T cells (Tregs), and M1 macrophages (**Figure 8B**). These patterns suggest an immunosuppressive tumor microenvironment (TME) within the high-risk group, potentially contributing to poorer prognostic outcomes. Parallel analyses within the GSE65904 dataset corroborated these findings, demonstrating increased infiltration of CD4^+^ memory resting T cells, activated natural killer (NK) cells, non-activated macrophages (M0), and M2 macrophages in the high-risk cohort, alongside decreased levels of plasma cells, CD8^+^ T cells, activated CD4^+^ memory T cells, γδ T cells, M1 macrophages, and resting dendritic cells (**Figures 8C, D**). These results elucidate the intricate interplay between the KMPAG signature and the immune landscape of SKCM, highlighting the role of macrophage polarization and T cell dynamics in influencing patient prognosis.

### 3.8 KMPAG signature predicts immunotherapy response in melanoma

**Figure 9:**
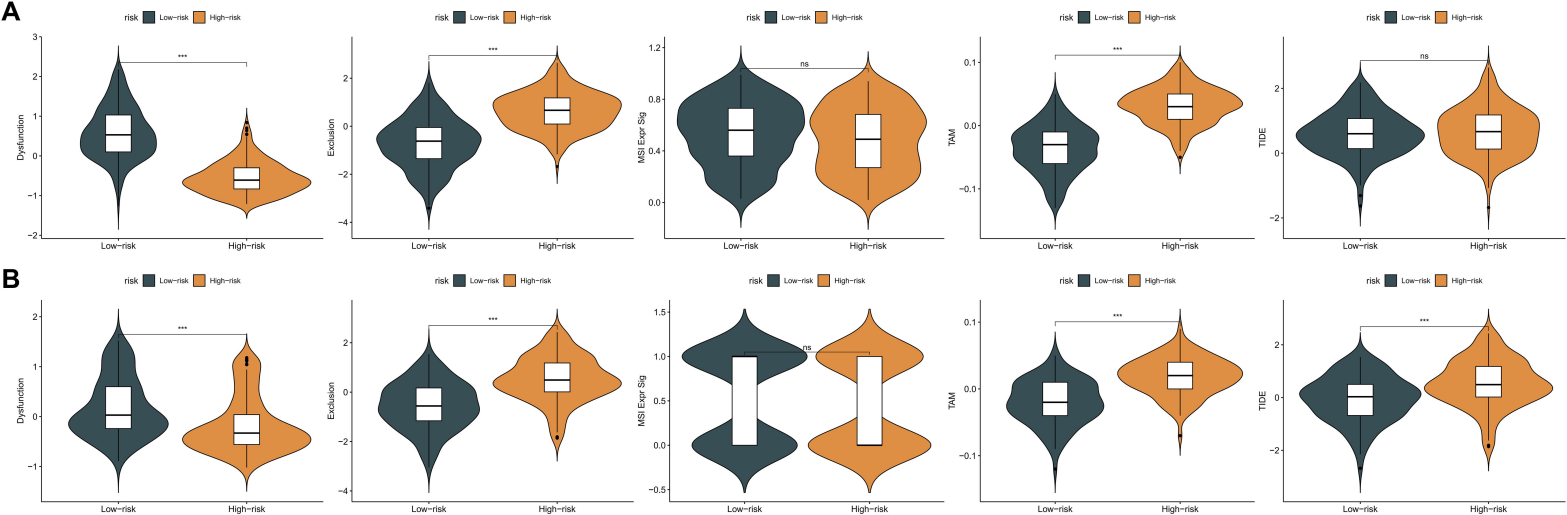
Evaluation of KMPAG signature association with immunotherapy response biomarkers. **(A)** Comparison of TIDE scores (T cell dysfunction, exclusion, M2 TAM) between KMPAG high- and low-risk groups in TCGA-SKCM. **(B)** Corresponding analysis in the GSE65904 cohort. **(C, D)** Validation attempt using clinical outcomes (PFS, Response Rate) in the external ICB-treated cohort GSE78220. ns: p > 0.05; ***p < 0.001.

Given the pivotal role of immune modulation in melanoma treatment, the predictive capacity of the KMPAG signature for immunotherapy response was evaluated using the Tumor Immune Dysfunction and Exclusion (TIDE) algorithm. This computational approach assesses mechanisms of immune evasion, including T cell dysfunction and exclusion, to predict responsiveness to immune checkpoint blockade (ICB) therapies. Analysis of the TCGA-SKCM dataset revealed that high-risk patients exhibited significantly elevated T cell exclusion scores and increased presence of tumor-associated macrophages (TAMs) with an M2 phenotype, while low-risk patients demonstrated lower levels of T cell dysfunction (**Figure 9A**). Similar trends were observed in the GSE65904 dataset, where high-risk cohorts showed higher T cell exclusion and TAM M2 scores, and lower T cell dysfunction scores (**Figure 9B**). Notably, no significant differences were detected in microsatellite instability (MSI) scores or overall TIDE scores between the risk groups. Given the TIDE prediction suggesting potential links between the KMPAG signature and immune evasion mechanisms, we sought to validate its predictive utility in the independent GSE78220 anti-PD1 treated melanoma cohort. Our analysis revealed that the KMPAG signature did not significantly predict clinical response (CR/PR vs SD/PD; P = 0.156) nor progression-free survival (P = 0.249) in this cohort.

These TIDE predictions suggest potential mechanisms of immune evasion in the high-risk group. However, interpreting these scores requires caution. For instance, the elevated T cell exclusion score in the high-risk group seems contradictory to the observed lower expression of CXCL10, a key chemoattractant for T cells, within this group. This discrepancy highlights the complexity of predicting immunotherapy response based solely on transcriptomic signatures and warrants further investigation into the interplay between different immune evasion mechanisms. Furthermore, the lack of significant predictive power in the GSE78220 cohort underscores the challenges in translating prognostic signatures into robust predictive biomarkers for ICB.

### 3.9 Differential drug sensitivity profiles in high- and low-risk melanoma patients

To explore the therapeutic implications of the KMPAG risk model, drug sensitivity analyses were performed using the pRRophetic algorithm, which predicts chemotherapeutic response based on gene expression profiles. A panel of 14 antineoplastic agents, encompassing both chemotherapeutic and targeted therapies, was selected for evaluation (**Supplementary Figures S6A–N**). High-risk patients demonstrated significantly increased predicted sensitivity to Bortezomib, Cisplatin, Dasatinib, Erlotinib, Gefitinib, Lapatinib, Lenalidomide, Methotrexate, Rapamycin (Sirolimus), and Temsirolimus (p < 0.05) (**Supplementary Figures S6A–J**). Conversely, low-risk patients exhibited heightened sensitivity to Bleomycin, Bryostatin 1, Doxorubicin, and Tipifarnib (**Supplementary Figures S6K–N**). These differential sensitivities suggest that the KMPAG signature could inform personalized therapeutic regimens, optimizing the selection of chemotherapeutic and targeted agents to enhance efficacy and mitigate adverse effects.

### 3.10 miRNA-mediated regulation of KMPAG signature genes in melanoma

Utilizing four comprehensive databases—miRDB, miRWalk, RNA22, and TargetScan—candidate miRNAs targeting CLEC4A, CXCL10, and LAT2 were systematically identified (**Supplementary Figures S7A–C**). To enhance the specificity of these interactions and minimize false positives, only miRNAs predicted by at least two databases were considered for further analysis. An informative upset plot was generated to visualize the overlapping miRNA interactions among the three hub genes across the four datasets, revealing distinct miRNA clusters associated with each gene (**Supplementary Figure S7D**). For CLEC4A, five miRNAs (hsa-miR-1206, hsa-miR-1266-3p, hsa-miR-488-5p, hsa-miR-5589-3p, and hsa-miR-6873-3p) were consistently predicted across all four databases, suggesting a robust regulatory role in modulating CLEC4A expression in melanoma. Similarly, CXCL10 was linked to fifteen miRNAs, including hsa-miR-34a-5p and hsa-miR-204-3p, while LAT2 was associated with nine distinct miRNAs such as hsa-miR-326 and hsa-miR-330-5p (**Supplementary Figure S7D**). Despite the comprehensive analysis, no single miRNA was found to concurrently target all three hub genes, indicating a gene-specific regulatory mechanism. These findings illuminate the complex regulatory networks governing gene expression in melanoma and underscore the significance of miRNA-mediated modulation in the context of KRAS signaling and macrophage biology.

### 3.11 Macrophage-mediated immune cell communication and its impact on tumor progression

**Figure 10:**
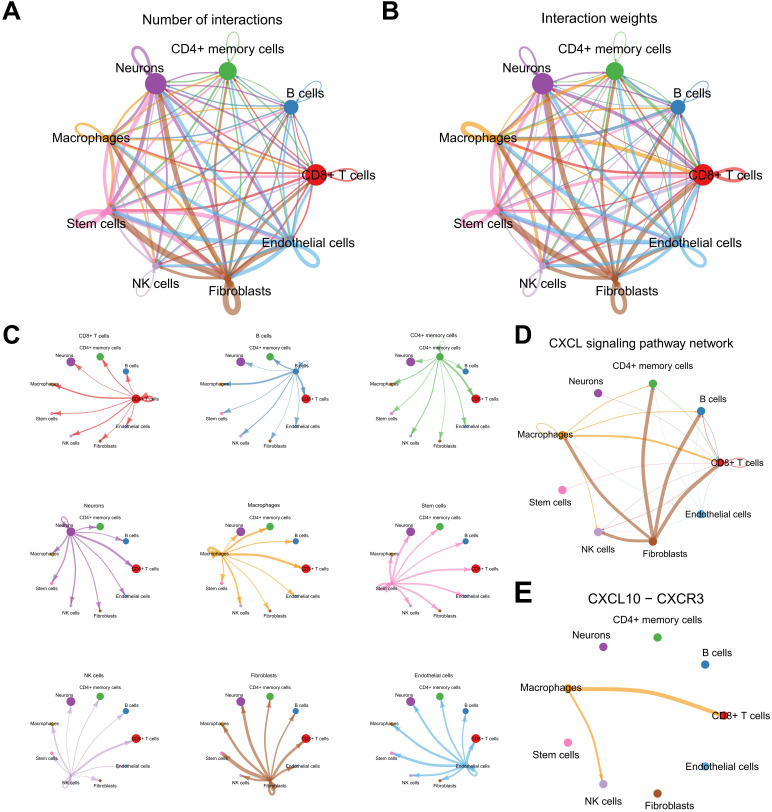
Analysis of the cell-cell communication network in the melanoma TME. **(A, B)** CellChat summary plots showing the frequency and weight of intercellular interactions. **(C)** Heatmap illustrating cell types as signal senders/receivers. **(D, E)** Network analysis highlighting the CXCL signaling pathway and key interactions involving macrophages.

To elucidate the intercellular communication dynamics within the SKCM tumor microenvironment, the CellChat algorithm was employed to construct and analyze ligand-receptor interaction networks among various cellular subsets (**Figures 10A, B**). The analysis revealed a highly interconnected network of ligand-receptor pairs, emphasizing the pivotal role of macrophages as both signal senders and receivers (**Figure 10C**). Notably, the Chemokine (C-X-C motif) Ligand (CXCL) signaling pathway emerged as a critical mediator of communication between macrophages and other immune cells, including CD8^+^ T cells and natural killer (NK) cells (**Figures 10D, E**).

Further investigation into the CXCL10-CXCR3 interaction demonstrated that macrophages predominantly express CXCL10, which engages the CXCR3 receptor on CD8^+^ T cells and NK cells, facilitating their recruitment and activation within the tumor microenvironment (**Supplementary Figure S8A**). Additionally, the CXCL16-CXCR6 axis was identified as another significant pathway, particularly in interactions between macrophages and CD8^+^ T cells, suggesting a complementary mechanism for immune cell trafficking and retention (**Supplementary Figure S8B**). Network centrality analyses underscored macrophages as central hubs within the communication network, orchestrating the spatial and functional organization of immune infiltrates (**Supplementary Figure S8C**). These intercellular interactions highlight the intricate balance between pro-inflammatory and immunosuppressive signals in SKCM, mediated by macrophage-derived chemokines, and underscore the potential of targeting these pathways to modulate immune responses and improve therapeutic outcomes.

### 3.12 CLEC4A expression and its functional role in melanoma cell proliferation and invasion

**Figure 11:**
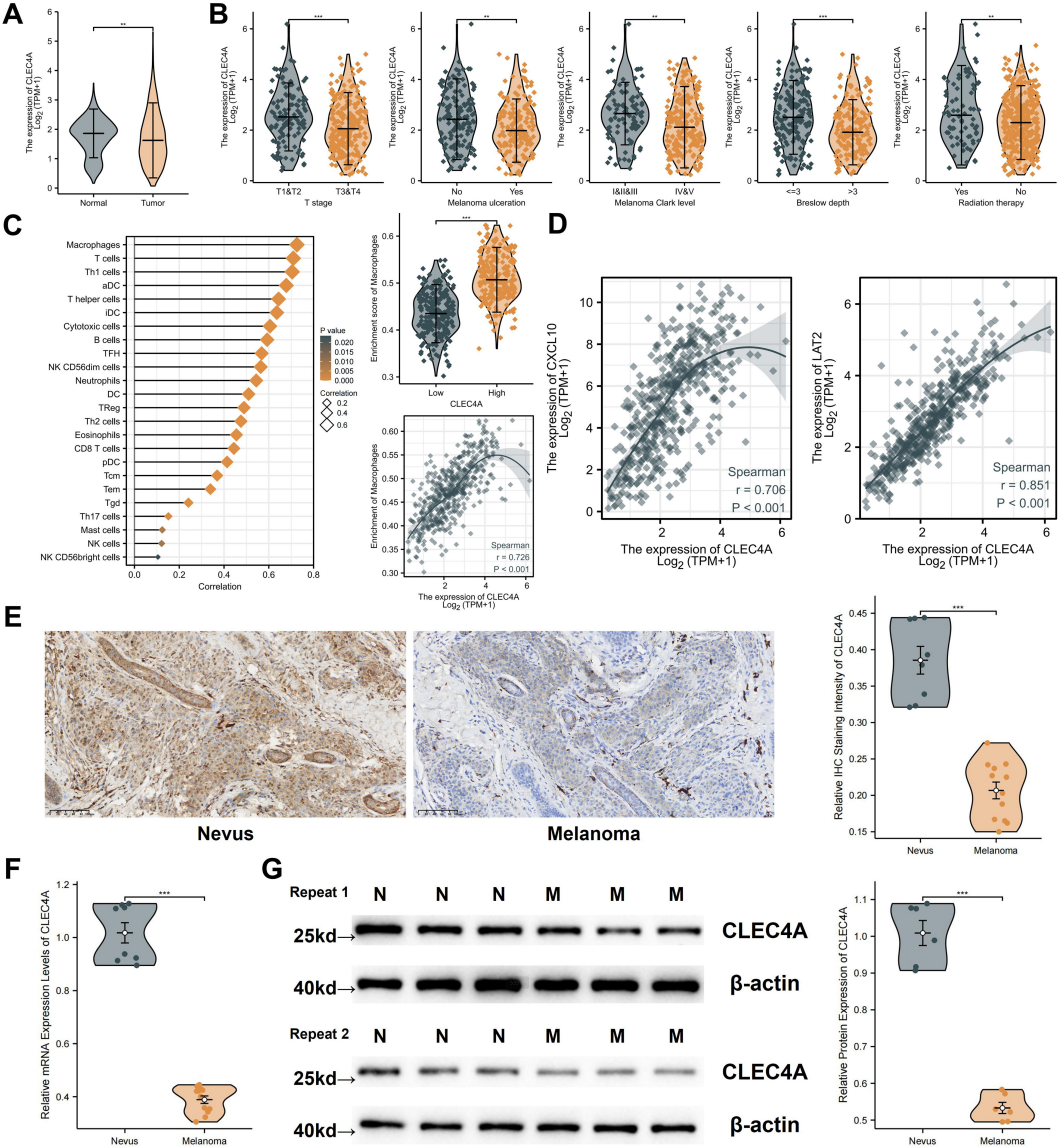
Expression analysis and clinical relevance of CLEC4A in melanoma. **(A)** CLEC4A expression comparison (TCGA-SKCM vs. GTEx). **(B)** CLEC4A expression across clinical groups in TCGA-SKCM. **(C)** Correlation of CLEC4A expression with immune infiltration. **(D)** Correlation of CLEC4A with CXCL10 and LAT2. **(E)** Representative IHC staining of CLEC4A in benign nevi vs. melanoma. **(F)** RT-qPCR analysis of CLEC4A mRNA levels in 8 benign nevi vs. 12 melanoma tissues. **(G)** Western blot analysis of CLEC4A protein expression in benign nevi [N] vs. melanoma tissues [M]. **p < 0.01; ***p < 0.001.

The expression and clinical significance of CLEC4A in melanoma were comprehensively evaluated by comparing data from TCGA-SKCM and GTEX-Skin datasets. Analysis revealed that CLEC4A expression is significantly lower in melanoma tissues compared to normal skin (**Figure 11A**). Further stratification within the TCGA-SKCM cohort indicated a negative correlation between CLEC4A expression and various clinical parameters, including T stage, presence of ulceration, Clark level of invasion, Breslow depth, and prior radiation therapy (**Figure 11B**). This suggests that reduced CLEC4A expression may serve as an indicator of disease progression in melanoma. The immune landscape within the tumor microenvironment was assessed, revealing a strong positive correlation (r = 0.726) between CLEC4A expression and macrophage infiltration (**Figure 11C**). Moreover, CLEC4A expression exhibited significant correlations with CXCL10 (r = 0.706) and LAT2 (r = 0.851), suggesting potential cooperative roles of these genes in melanoma (**Figure 11D**). Immunohistochemical analysis of melanoma and benign nevi further corroborated these findings, showing a marked decrease in CLEC4A protein levels in malignant melanoma tissues (**Figure 11E**). RT-qPCR results demonstrated that CLEC4A mRNA expression was significantly lower in melanoma tissues (n=12) compared to benign nevi (n=8) (**Figure 11F**, P < 0.001). Consistent with the mRNA levels and IHC staining, Western blot analysis also revealed a notable reduction in CLEC4A protein expression in melanoma tissues compared to benign nevi, with this pattern being reproducible across replicates (**Figure 11G**). These data collectively confirm the downregulation of CLEC4A in melanoma at both mRNA and protein levels.

**Figure 12:**
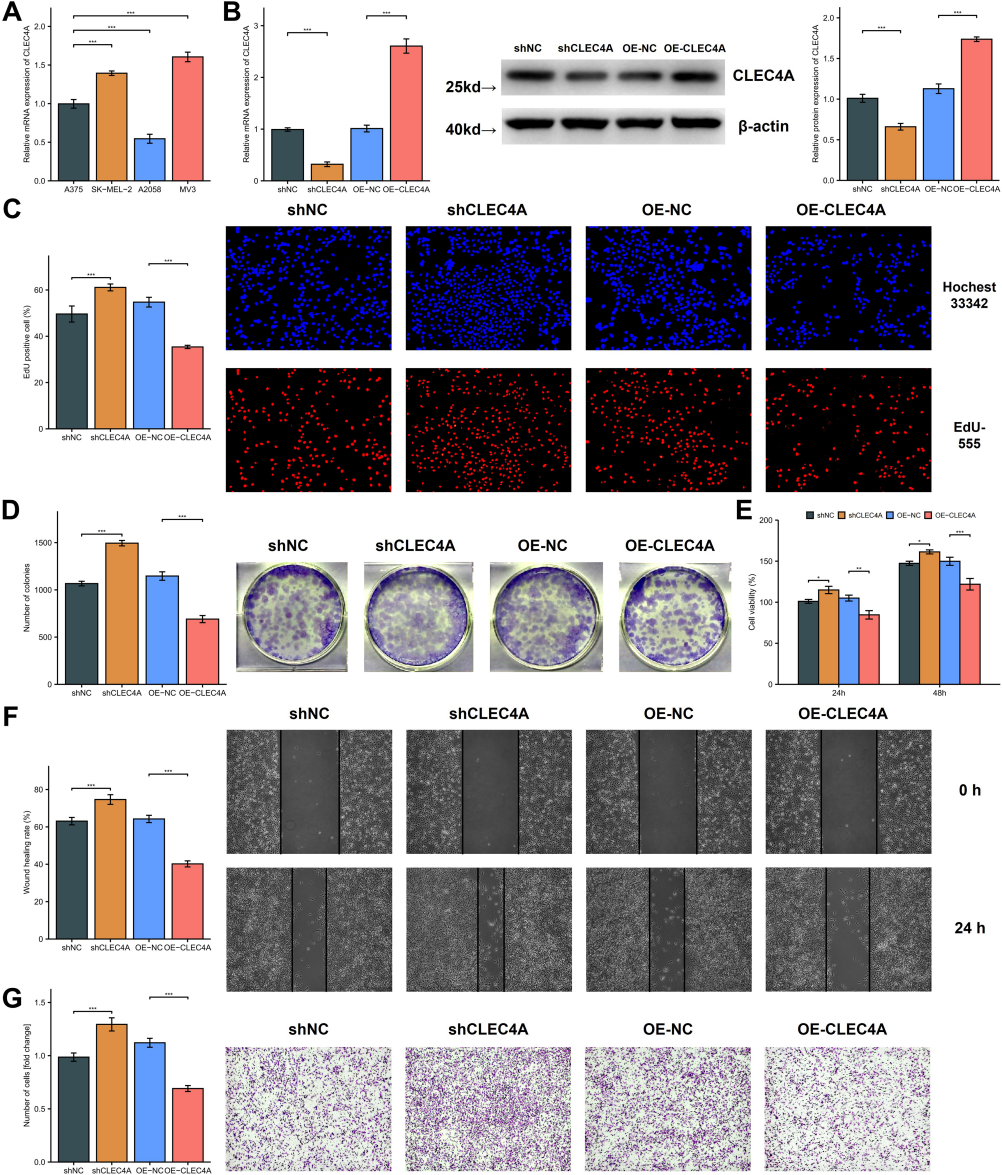
Functional investigation of CLEC4A’s role in melanoma cell behavior. **(A)** CLEC4A mRNA expression in melanoma cell lines. **(B)** Verification of CLEC4A knockdown/overexpression in A375 cells. **(C-E)** Proliferation assays (EdU, colony formation, CCK-8). **(F, G)** Migration and invasion assays (wound healing, Transwell). *p < 0.05; **p < 0.01; ***p < 0.001.

A preliminary expression profiling across various melanoma cell lines revealed differential CLEC4A expression patterns, with the A375 cell line exhibiting relatively lower CLEC4A levels when compared to SK-MEL-2 and MV3, yet higher levels compared to A2058 (**Figure 12A**). To explore the functional implications of CLEC4A expression in melanoma, a series of cellular assays were performed using A375 melanoma cells, which were transduced with lentiviral constructs for CLEC4A knockdown and overexpression. The successful modulation of CLEC4A expression was confirmed by quantitative PCR and Western blot analysis (**Figure 12B**). A decrease in CLEC4A expression was associated with enhanced cellular proliferation, as indicated by the EdU, colony formation, and CCK-8 assays (**Figures 12C-E**). These findings suggest that CLEC4A may act as a suppressor of melanoma cell growth. Additionally, functional assays evaluating cell migration and invasion, including the wound-healing and Transwell assays, demonstrated a marked increase in both migratory and invasive capabilities following CLEC4A knockdown (**Figures 12F, G**). Together, these results suggest that CLEC4A plays a critical role in regulating melanoma cell motility and invasiveness, functioning as a potential molecular brake in melanoma progression.

## 4 Discussion

Efforts to elucidate the molecular hallmarks of melanoma have long focused on canonical mutations in genes such as BRAF and NRAS (2, 3). Nonetheless, subsets of melanomas appear to engage the KRAS signaling cascade in ways that may synergize or converge with these established oncogenic pathways (14, 67). The integrative analyses described herein have provided evidence suggesting that KRAS-related processes could underpin tumor progression, modulate immune cell infiltration, and confer distinctive biological properties on melanoma cells (9, 68). Although the overall mutation frequency of KRAS in cutaneous melanoma may be less pronounced than that of BRAF or NRAS, the current findings reinforce the concept that KRAS pathway activation is sufficiently relevant to warrant deeper examination (14). The identification of genes linked to patient outcomes and immune components in the tumor microenvironment further underscores the notion that KRAS signaling does not operate in isolation but rather interconnects with immunological mediators, including macrophage-dependent processes (28). These observations complement prior studies indicating that KRAS pathway alterations are associated with aggressive phenotypes in certain melanomas (15, 69), although additional mechanistic work is essential to validate any direct causative links. It is important to acknowledge that direct activating mutations in KRAS are relatively infrequent in cutaneous melanoma (<5% in TCGA) compared to BRAF or NRAS mutations. Our focus on a ‘KRAS-Macrophage-Associated’ signature stems primarily from GSEA results indicating enrichment of KRAS signaling pathways associated with prognosis, rather than a reliance on KRAS mutation status itself. This suggests that downstream effectors of the KRAS pathway, or related RAS-MAPK signaling components, might be activated through other mechanisms (e.g., upstream receptor tyrosine kinase activity, feedback loops, or crosstalk from other pathways like PI3K/AKT) in a larger subset of melanomas, contributing to the observed prognostic associations and TME modulation. Future studies should explore these alternative mechanisms of KRAS pathway activation in melanoma subtypes lacking direct KRAS mutations.

Beyond its direct influence on tumor cells, KRAS signaling appears to shape the immunological landscape of melanoma through an intricate web of cytokines, chemokines, and other regulatory factors (16). The macrophage-centric gene signature described in this study, comprising CLEC4A, CXCL10, and LAT2, furnishes robust evidence that macrophages can vary widely in their functional states within melanoma lesions (30). These shifts may correspond to different phenotypic extremes, such as proinflammatory M1-like macrophages that support antitumor immunity or immunosuppressive M2-like macrophages that facilitate tumor expansion and immune evasion (19). The KMPAG signature, in particular, highlights genes that potentially modulate both macrophage polarization and tumor cell behavior, suggesting that complex crosstalk between KRAS-activated cells and infiltrating macrophages fuels disease progression (20, 31). The detection of strong correlations between the KMPAG signature and clinical features, including survival outcomes and immunotherapy responses, strengthens the argument that macrophages serve as more than mere bystanders in the melanoma microenvironment (31). Instead, these cells appear to be pivotal arbiters of immune surveillance, acting under the orchestration of oncogenic signals to either enhance or dampen the local immune response. By distilling these intricate interactions into a pragmatic gene panel, the analysis widens the scope for personalized therapies that target macrophage-specific pathways while recognizing the heterogeneity intrinsic to melanoma biology (18). Such precision-driven approaches may exploit signals that tip the macrophage balance toward an M1-like state, thereby bolstering tumor-directed cytotoxicity, or disrupt KRAS-dependent immunosuppression in synergy with established immunotherapies (19).

The capacity of the KRAS-Macrophage Prognostic Associated Gene (KMPAG) signature to delineate high-risk and low-risk melanoma cohorts underscores its translational potential for refining clinical decision-making (28). The observation that diminished expression of CLEC4A, CXCL10, and LAT2 aligns with more aggressive disease provides a direct biomarker-based framework to categorize patients who may require closer surveillance or intensified therapeutic regimens (30). Regarding CLEC4A specifically, its reduced expression in melanoma tissues compared to benign nevi, a trend initially suggested by our analysis of transcriptomic databases and confirmed by immunohistochemical staining of tissue sections. Our subsequent RT-qPCR and Western blot analyses on clinical tissue samples decisively demonstrated this downregulation at both the mRNA and protein levels. This concordance across multiple independent detection methodologies significantly strengthens the reliability of CLEC4A’s diminished expression as a consistent molecular feature in melanoma, reinforcing its potential role and prognostic significance. In parallel, the association of the KMPAG signature with specific immune cell profiles—particularly those involving tumor-infiltrating macrophages—offers a nuanced lens through which clinicians and researchers can anticipate immunotherapy responses (48). This signature may help distinguish tumors more apt to benefit from immune checkpoint inhibitors from those prone to resistance (7), thus enabling the rational selection of additional or alternative treatment strategies, including targeted therapies against pro-tumoral macrophage functions. High-risk patients showed significantly lower predicted IC50 values (indicating higher sensitivity) for several agents including Bortezomib, Cisplatin, and mTOR inhibitors like Rapamycin (Sirolimus) and Temsirolimus according to the pRRophetic algorithm. However, these in silico predictions should be interpreted with caution. For example, the predicted sensitivity to mTOR inhibitors in the high-risk group contrasts with some preclinical evidence suggesting that KRAS-driven cancers, potentially relevant to pathways enriched in our high-risk group, might exhibit resistance to PI3K/mTOR pathway inhibition (70). Therefore, these computational drug sensitivity findings represent hypotheses requiring direct experimental validation rather than definitive clinical recommendations. The prospect of coupling the KMPAG signature with genomic profiling of other prevalent melanoma driver mutations (e.g., BRAF, NRAS) adds another layer of precision medicine, as it may reveal composite molecular phenotypes that inform unique susceptibility or resilience to various therapeutic interventions (26).

We acknowledge that our study primarily relies on transcriptomic correlations and GSEA to infer the involvement of KRAS signaling, rather than direct mechanistic experiments. While our in silico analysis did not show a direct correlation between KRAS mutation status and the KMPAG signature in the TCGA cohort, the GSEA results consistently point towards an enrichment of KRAS pathway activity associated with prognosis. Methodologically, our prognostic model was developed using LASSO regression. While effective for feature selection in high-dimensional data, we acknowledge that relying solely on LASSO might introduce selection bias, and comparing its performance with other regularization methods like Ridge or Elastic Net, or employing feature importance techniques like permutation testing, could further strengthen the model’s robustness. Additionally, while validated in an independent GEO cohort, the KMPAG signature’s prognostic utility and predictive power for immunotherapy response ideally require validation in prospective studies or well-annotated retrospective cohorts specifically treated with modern checkpoint inhibitors. The model’s performance should also be evaluated considering key AJCC criteria like Breslow thickness, which was not consistently available across all datasets used. Our definition of macrophage subpopulations relied on unsupervised clustering of scRNA-seq data (resolution=0.5). While we supplemented this with M1/M2 scoring and pathway analysis, we acknowledge that these computationally derived clusters may not perfectly map onto functionally distinct, biologically validated macrophage states like canonical M1/M2 or specific TAM subtypes described in other contexts. Validation using orthogonal methods, such as flow cytometry based on surface marker expression or spatial transcriptomics to assess localization relative to tumor cells, is necessary. Furthermore, the functional implications suggested by pathway enrichment (e.g., RAGE signaling in cluster 0) remain speculative without targeted experimental validation, such as using RAGE inhibitors or knockdowns in macrophage models.

An important consideration is the prevalence of BRAF mutations (particularly V600E) in cutaneous melanoma and its potential interplay with the KRAS-associated pathways investigated here. Our experimental validation utilized the A375 cell line, which harbors the BRAF V600E mutation. While this mutation is a strong driver of melanoma proliferation, our findings demonstrate that modulating CLEC4A expression still significantly impacted proliferation, migration, and invasion in this BRAF-mutant context. This suggests that CLEC4A exerts functions that are, at minimum, additive to or partially independent of the primary BRAF V600E oncogenic signal in these cells. Furthermore, our KMPAG signature was derived and validated using large patient cohorts (TCGA-SKCM, GSE65904) that inherently include a mixture of mutational backgrounds (BRAF-mutant, NRAS-mutant, KRAS-mutant, wild-type, etc.). The signature’s prognostic significance across these heterogeneous cohorts, combined with GSEA results pointing to broader KRAS pathway activity enrichment (which may be influenced by factors beyond direct KRAS mutations), supports its relevance beyond a specific mutation type.

Although the current study provides valuable insights into KRAS-associated immunomodulation and the prognostic relevance of macrophage biology in melanoma, several limitations warrant acknowledgment. First, the analysis largely relied on transcriptomic datasets and *in vitro* assays, which may not fully capture the dynamic complexities of *in vivo* tumor–immune interactions (26, 67). While the KMPAG signature was successfully validated in the independent GSE65904 cohort, its generalizability would be further strengthened by testing in additional large, well-characterized melanoma cohorts. Our search for suitable public datasets with available expression data for all three signature genes and associated long-term survival information yielded limited options beyond GSE65904. Validation in larger prospective patient cohorts, ideally with well-curated clinicopathologic data and longitudinal follow-up, is essential to solidify the clinical utility of the KMPAG signature. Second, the singular focus on KRAS-centric pathways, although justified by emerging evidence of KRAS involvement in some melanomas, may overlook synergistic or compensatory signaling through parallel oncogenic axes such as those governed by BRAF, NRAS, or PI3K-AKT-mTOR (68). Investigating the additive or interacting roles of these pathways may uncover novel combination strategies capable of overcoming resistance.

Another point requiring careful consideration is the apparent paradox concerning CXCL10. While lower CXCL10 expression is part of our high-risk signature (associated with poor OS and higher T cell exclusion via TIDE), CXCL10 is generally considered a potent chemoattractant for cytotoxic T lymphocytes and NK cells, and its higher expression has often been linked to favorable responses to immunotherapy in melanoma (30, 71). The lower CXCL10 in our high-risk group might reflect a specific immunosuppressive TME state where mechanisms suppressing CXCL10 production dominate, or where T cell exclusion is driven by factors overriding chemokine gradients. Alternatively, the prognostic association might reflect complex dynamics where initial high CXCL10 attracts T cells, but sustained inflammation or other factors in the high-risk TME lead to T cell exhaustion and dysfunction (partially supported by TIDE trends), ultimately resulting in poor outcome despite the chemokine’s presence or eventual downregulation. The precise role and regulation of CXCL10 within the context of the KMPAG signature and different risk strata warrant further mechanistic investigation.

While our study provides comprehensive bioinformatic analyses linking the KMPAG signature to melanoma prognosis and the tumor microenvironment, we acknowledge that the experimental validation presented has limitations in scope. Our experimental validation focused on the functional role of CLEC4A through knockdown and overexpression in the A375 melanoma cell line. While these experiments demonstrated a significant impact on tumor cell proliferation, migration, and invasion, suggesting a tumor-suppressive role. Firstly, the validation was tumor-centric. Given that our bioinformatic analyses, particularly scRNA-seq, indicate that CLEC4A is highly expressed in macrophages within the TME, its function within these immune cells remains uninvestigated in our study. Future experiments using macrophage cell lines (THP-1 differentiated macrophages) or primary human macrophages with CLEC4A modulation are crucial to understand its role in macrophage polarization, phagocytosis, or antigen presentation in the context of melanoma. Secondly, the functional roles of the other two KMPAG signature genes, CXCL10 and LAT2, were not experimentally assessed. CXCL10 is a well-known chemokine; validating its chemotactic effect on immune cells towards melanoma cells or within the TME using Transwell migration assays would be essential. Similarly, LAT2 is involved in amino acid transport; investigating its role in metabolic crosstalk between tumor cells and immune cells, potentially under nutrient stress conditions like glutamine deprivation, would provide valuable mechanistic insights. Therefore, while our CLEC4A findings in A375 cells provide initial support, comprehensive validation requires exploring all three genes’ functions, particularly within the relevant immune cell compartments like macrophages.

Finally, the generalizability of the KMPAG signature across different melanoma subtypes needs consideration. Cutaneous melanoma is heterogeneous, influenced by factors like UV mutation signature burden and underlying driver mutations. Our model was developed and validated using large cohorts (TCGA, GSE65904) likely encompassing a mixture of subtypes. However, we did not specifically assess the performance of the KMPAG signature within distinct molecular or etiological subtypes (e.g., NF1-mutant melanomas, which can exhibit RAS pathway activation, or acral vs. mucosal melanomas). Future work should investigate whether the prognostic value and biological implications of the KMPAG signature differ across these subtypes, potentially leading to more refined, subtype-specific prognostic tools.

## 5 Conclusion

The multifaceted investigation integrating KRAS pathway perturbations, macrophage-related gene expression, and immune landscape analyses has culminated in a more comprehensive understanding of melanoma’s complex biology. The evidence presented supports a pivotal function for signaling associated with the KRAS pathway and macrophage infiltration in shaping tumor progression, immune evasion, and therapeutic responsiveness. The three-gene KRAS-Macrophage Prognostic Associated Gene (KMPAG) signature, encompassing CLEC4A, CXCL10, and LAT2, emerges as a robust prognostic tool that correlates with distinct clinical and immunological phenotypes in melanoma cohorts. Notably, the convergence of reduced KMPAG gene expression, immunosuppressive macrophage infiltration, and adverse clinical features underscores the importance of targeted interventions aimed at reversing these tumor-promoting conditions. Moreover, mechanistic evidence indicating a tumor-suppressive role for CLEC4A suggests that fine-tuning key macrophage-associated genes may complement existing immunotherapies. Although additional prospective and mechanistic research is essential to validate and refine these findings, the results collectively reaffirm the therapeutic potential of targeting the interplay between oncogenic drivers and the tumor immune microenvironment.

## Data Availability

The original contributions presented in the study are included in the article/**Supplementary Material**. Further inquiries can be directed to the corresponding authors.
