# Supplementary material for: Identification and validation of a KRAS-macrophage-associated gene signature as prognostic biomarkers and potential therapeutic targets in melanoma
Source: Front Immunol. 2025 Jun 18;16:1566432. doi: 10.3389/fimmu.2025.1566432 (PMC12213886; doi:10.3389/fimmu.2025.1566432)
Supplement: Supplementary file 1 [file Table1.docx]

Supplementary Material

# Supplementary Figures





**Figure S1**. Analysis of Marker Gene Expression Levels to Identify Immune Cell Clusters. Depicts the expression profiles of key immune-related marker genes used to categorize and demarcate distinct immune cell clusters. Higher or lower expression intensities guide the classification of T lymphocytes, B lymphocytes, macrophages, and additional subsets that shape the tumor microenvironment.


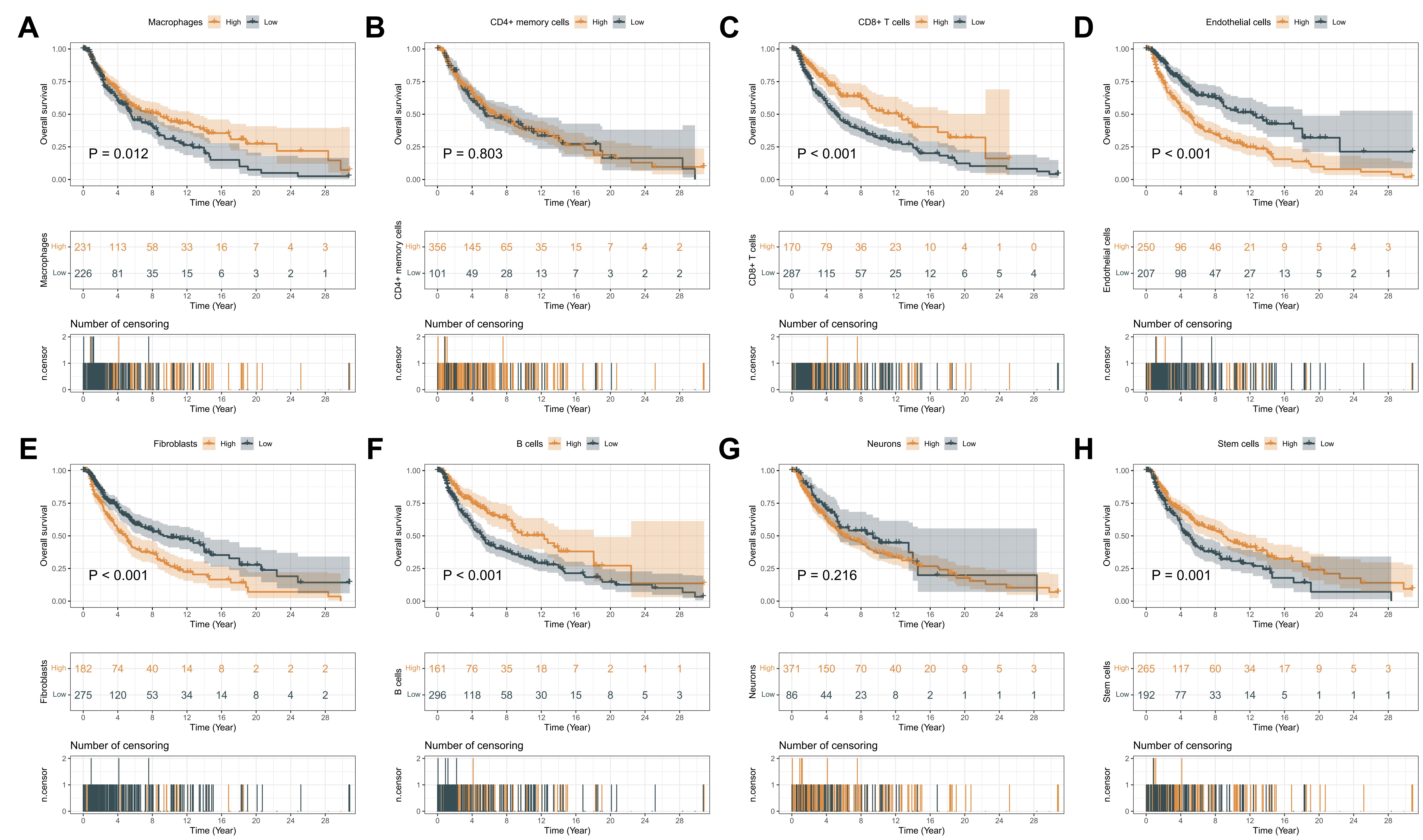


**Figure S2**. Association between Immune Cell Infiltration Levels and Overall Survival in TCGA-SKCM Patients. (A-H) Kaplan-Meier survival curves stratified by high vs. low ssGSEA-derived scores for various immune cell subpopulations.


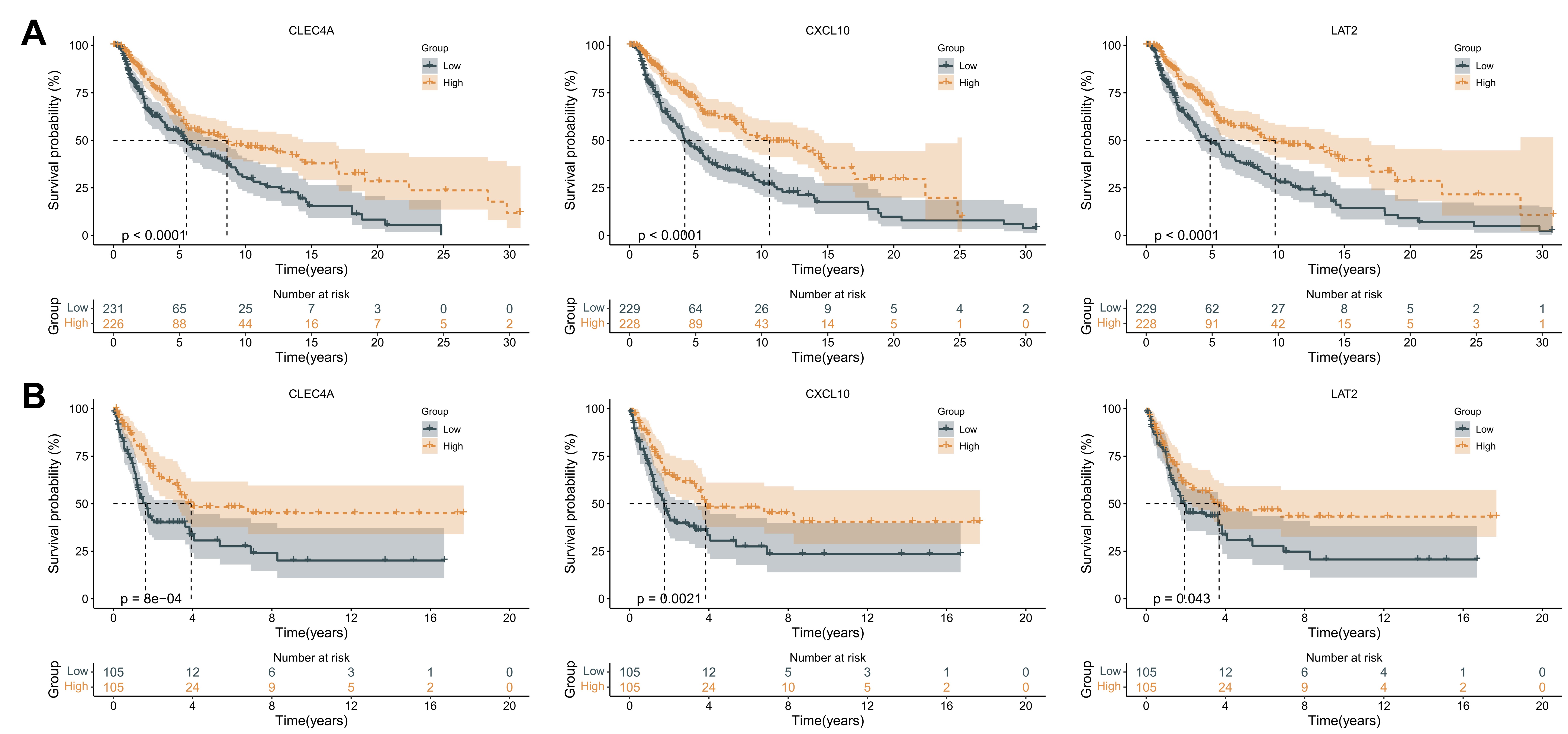


**Figure S3**. Survival Analysis Based on Individual KMPAG Signature Gene Expression. Kaplan-Meier OS curves comparing high vs. low expression groups for CLEC4A, CXCL10, and LAT2 in (A) TCGA-SKCM and (B) GSE65904 cohorts.


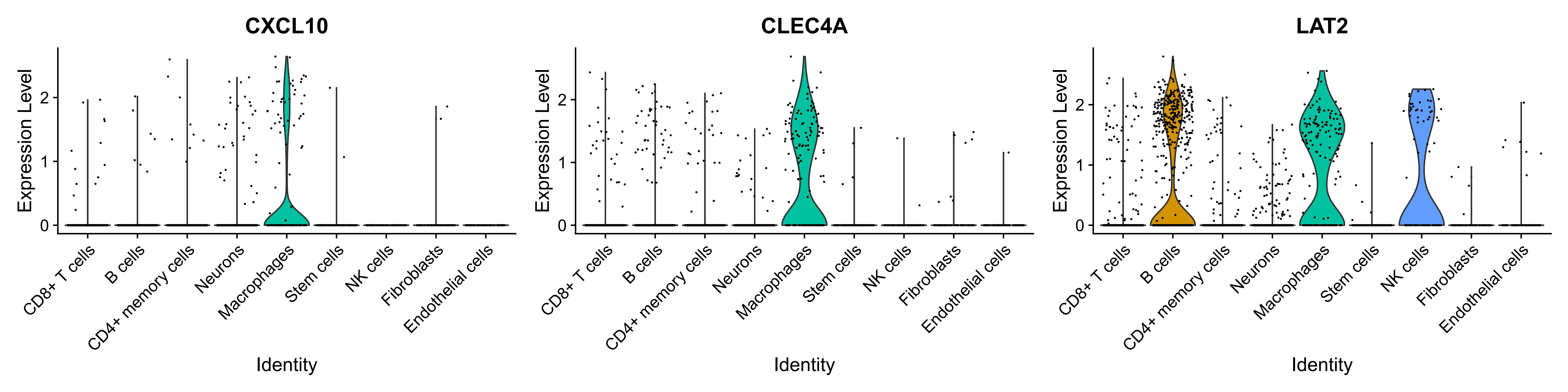


**Figure S4**. Expression Levels of KMPAG Signature Genes in Cell Subpopulations. Shows the distributions of CLEC4A, CXCL10, and LAT2 expression across various cellular subpopulations, providing insight into how the three-gene KRAS-Macrophage Prognostic Associated Gene (KMPAG) signature manifests within different lineage or functional compartments of the tumor.


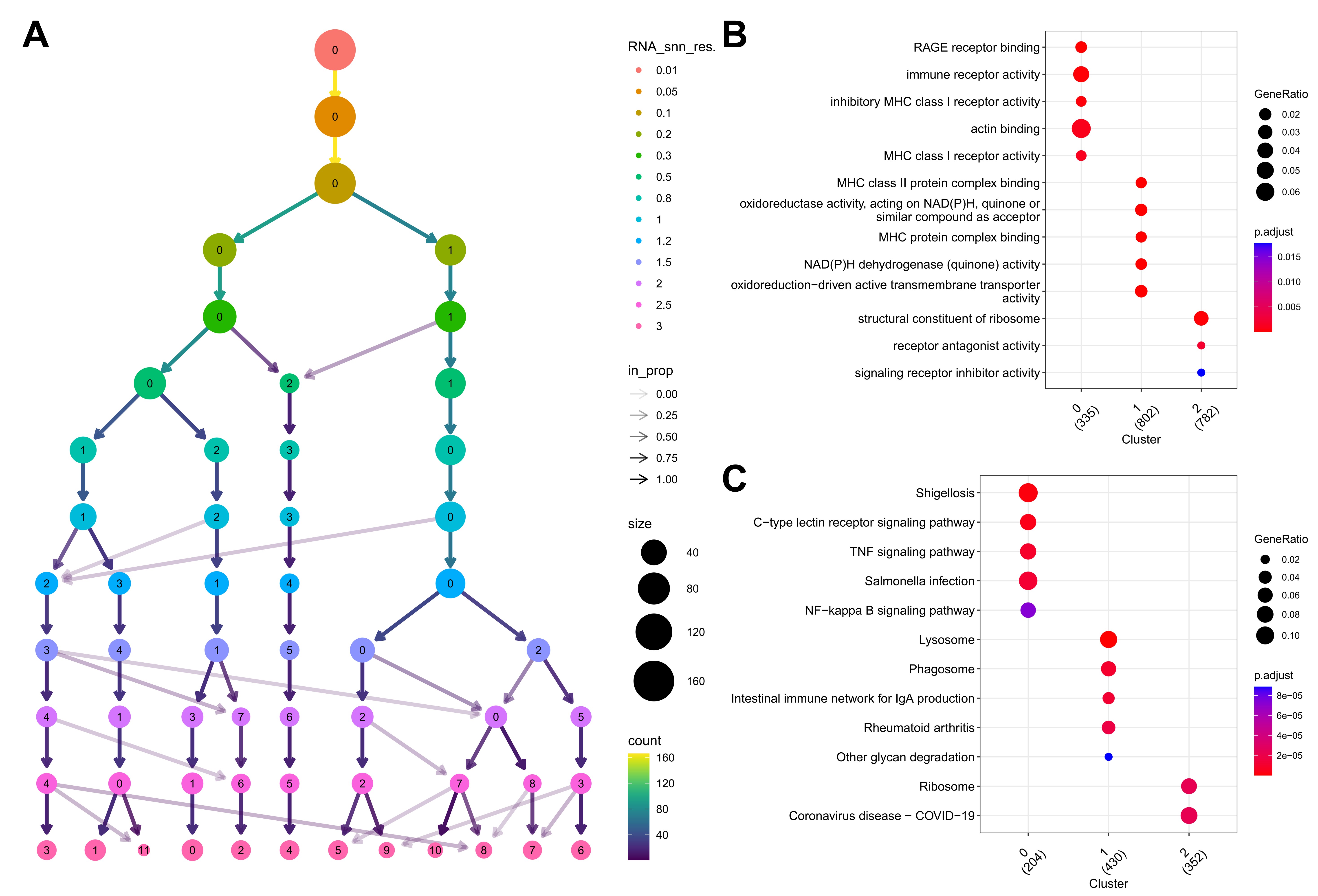


**Figure S5**. Reclustering Analysis of the Macrophage Subpopulation. (A) Clustertree depicting the resolution selection process for macrophage subclusters. (B) GO-based functional annotations indicating the biological processes enriched in each macrophage cluster. (C) KEGG pathway analysis highlighting metabolic or immunoregulatory cascades associated with these subclusters.


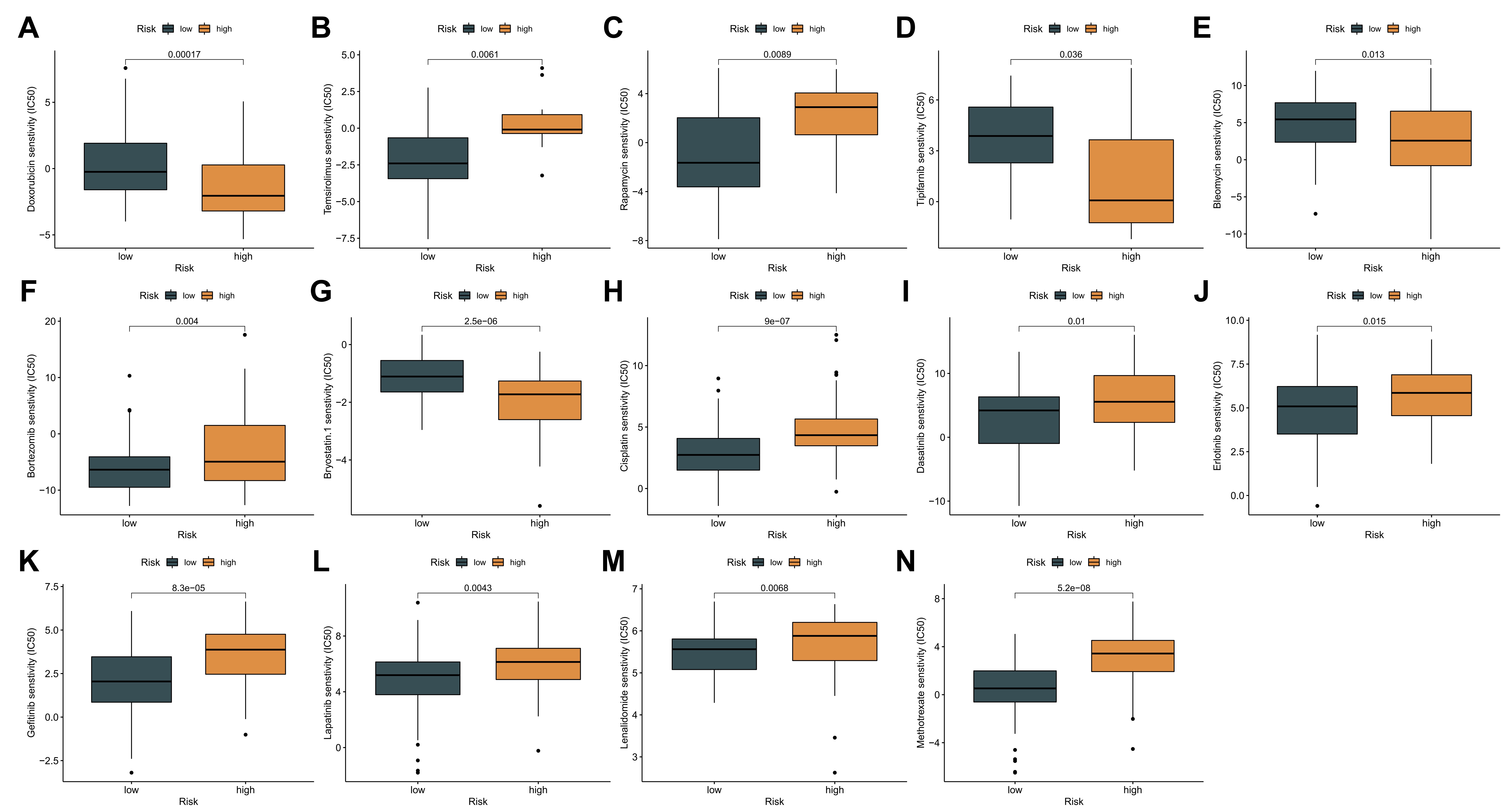


**Figure S6**. Predicted Drug Sensitivity Profiles Associated with KMPAG Risk Groups. Box plots showing predicted IC50 values for various agents (A-N) in high- vs. low-risk TCGA-SKCM patients using the pRRophetic algorithm.


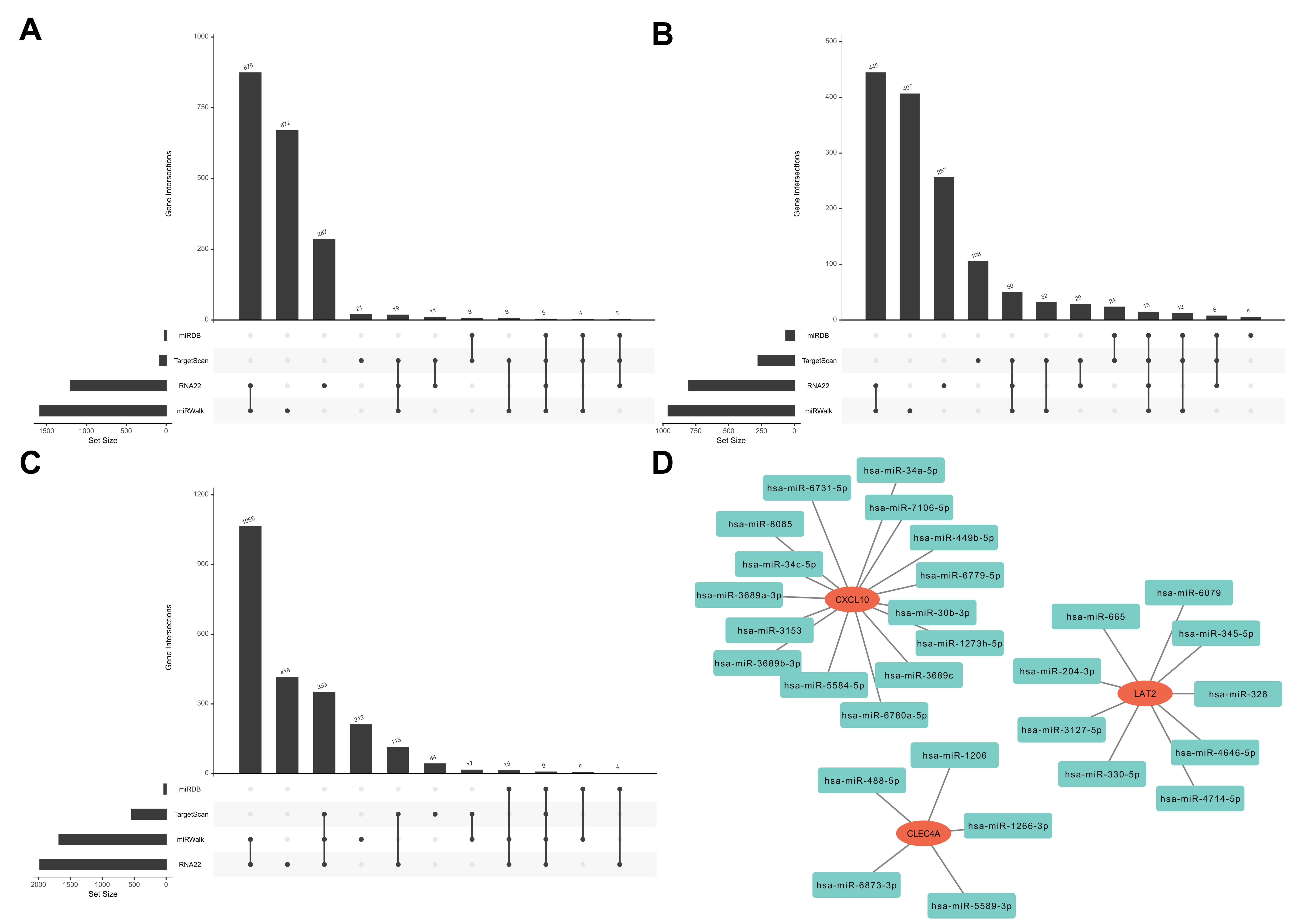


**Figure S7**. Predicted miRNA Regulatory Network Targeting KMPAG Hub Genes. (A–C) Upset plots summarizing miRNA predictions for each KMPAG gene across four databases. (D) Cytoscape visualization of the integrated miRNA-mRNA network for miRNAs predicted by at least two databases.


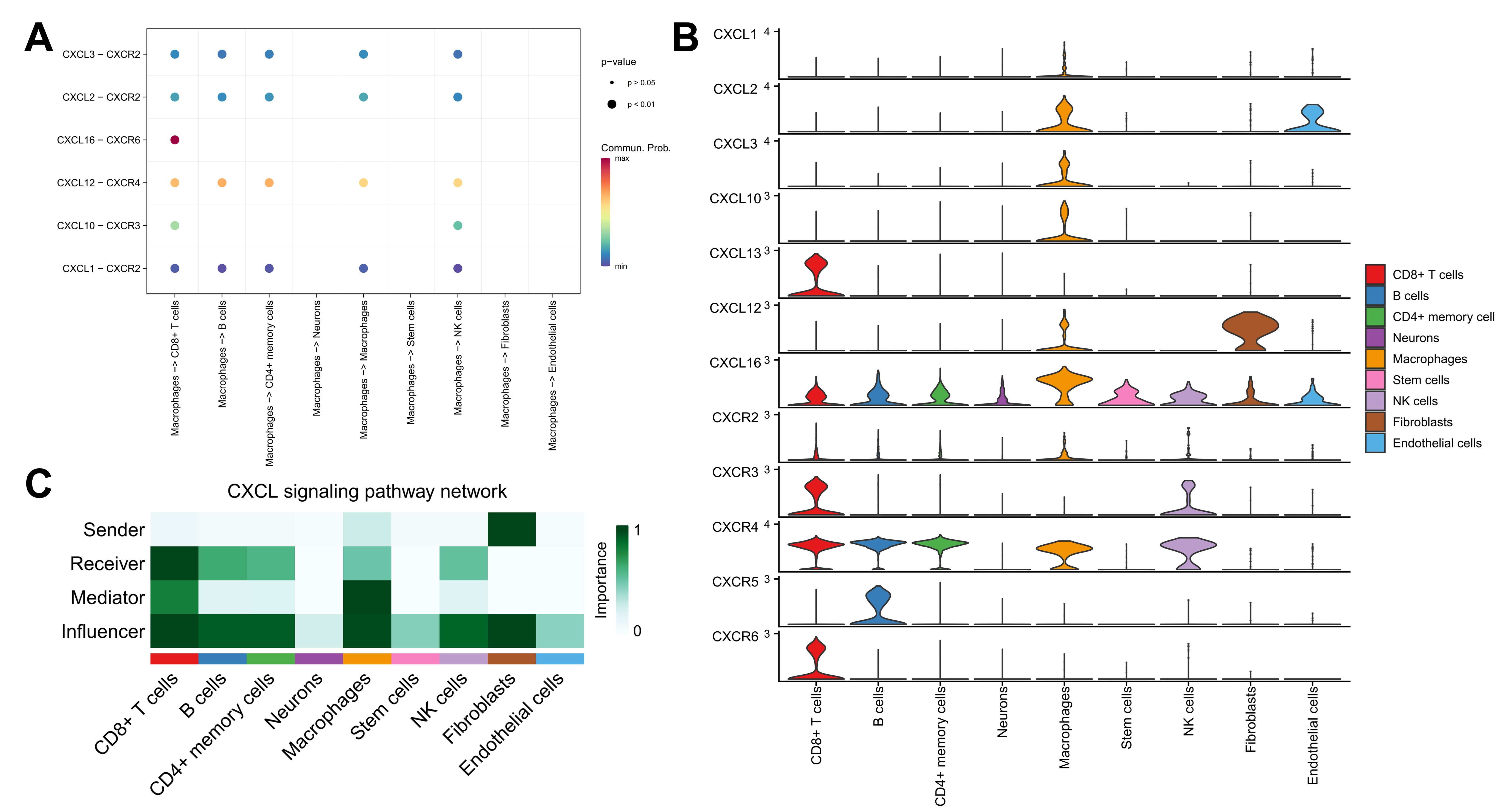


**Figure S8**. Detailed Analysis of Ligand-Receptor Pairs within the CXCL Signaling Pathway. (A) Contribution plots for CXCL10-CXCR3 and CXCL16-CXCR6 interactions. (B) Violin plots showing expression of key ligands/receptors across cell types. (C) Network centrality analysis for the CXCL pathway.

# Supplementary Table

**Table S1.** Critical Parameters for Key Analytical Methods

| **Analysis Method** | **Parameter** | **Value** | **Justification** |
| --- | --- | --- | --- |
| Seurat Clustering (Initial) | Resolution | 1.5 | Optimal cluster separation based on silhouette analysis |
| Seurat Clustering (Macrophages) | Resolution | 0.5 | Balanced cellular heterogeneity with biological coherence |
| LASSO Regression | Lambda (λ) | 0.0382 | Minimized cross-validation error in 10-fold CV |
| CellChat | Interaction Probability Threshold | 0.05 | Balance between sensitivity and specificity for interactions |
| CellChat | Permutation Test p-value | < 0.05 | Statistical significance threshold for interactions |
| TIDE Analysis | Dysfunction Score Threshold | 0 | Standard threshold as recommended by creators |
| DEG Analysis | log2FC Threshold | 1.0 | Biological significance threshold |
| DEG Analysis | Adjusted p-value | 0.05 | Statistical significance with FDR correction |
| Gene Set Enrichment | FDR q-value | 0.25 | Standard threshold for GSEA |
| Risk Score Stratification | Cutoff Method | Maximized Log-rank Statistic | Optimal separation of survival curves |
