# Supplementary figures and images for: Identification and validation of a KRAS-macrophage-associated gene signature as prognostic biomarkers and potential therapeutic targets in melanoma
Source: Front Immunol. 2025 Jun 18;16:1566432. doi: 10.3389/fimmu.2025.1566432 (PMC12213886; doi:10.3389/fimmu.2025.1566432)

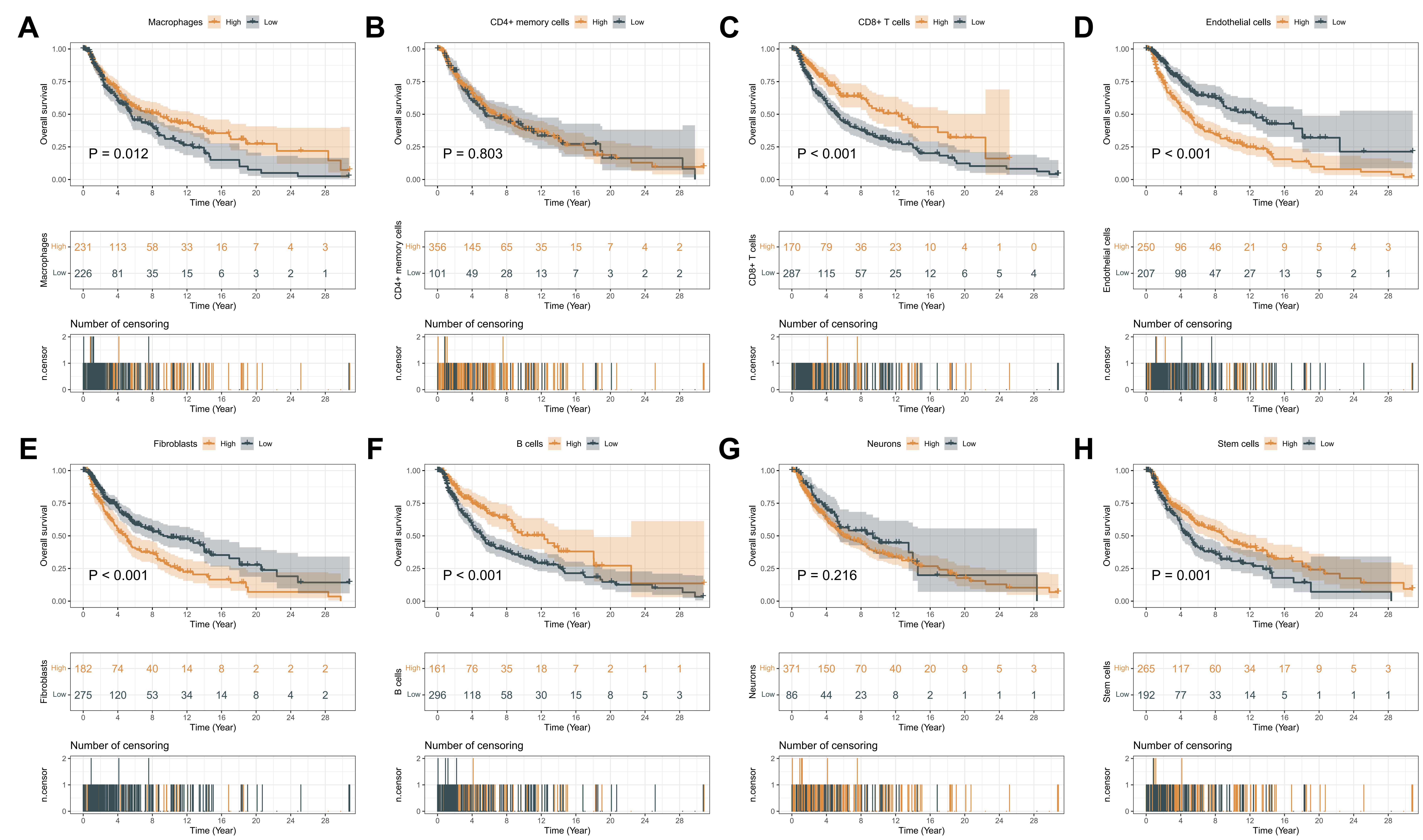

Supplement: Supplementary file 3 [file Image2.jpeg]

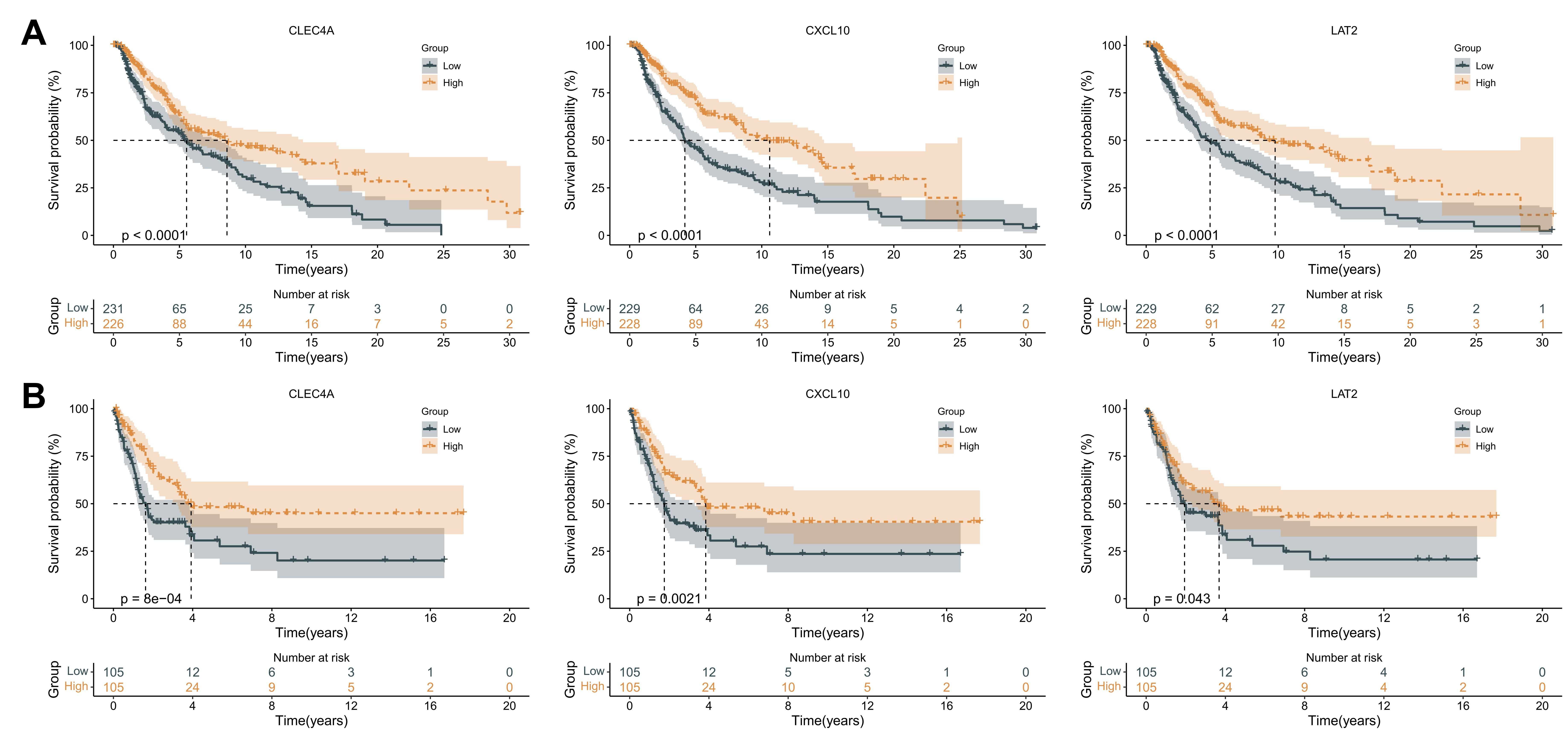

Supplement: Supplementary file 4 [file Image3.jpeg]

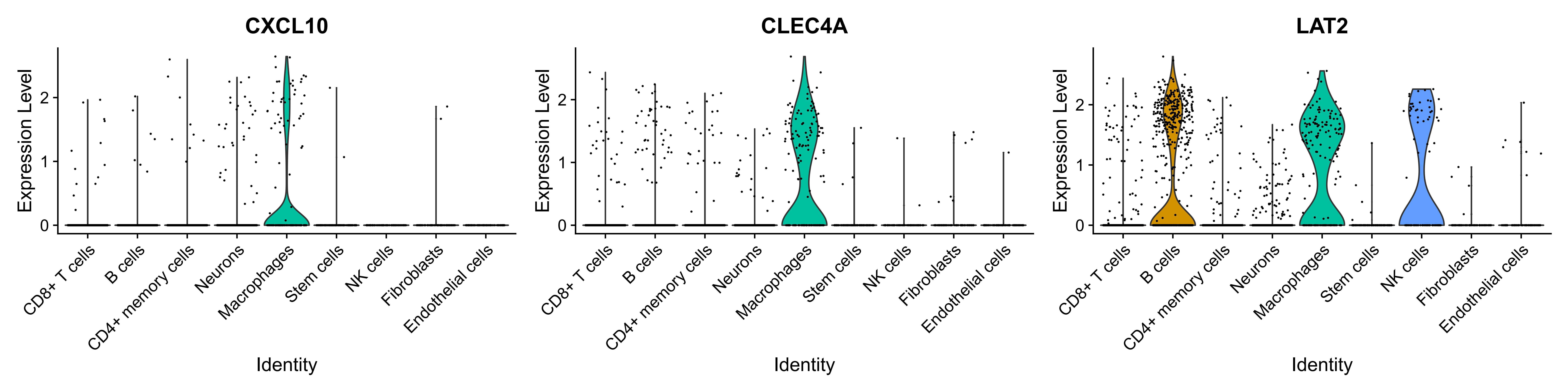

Supplement: Supplementary file 5 [file Image4.jpeg]

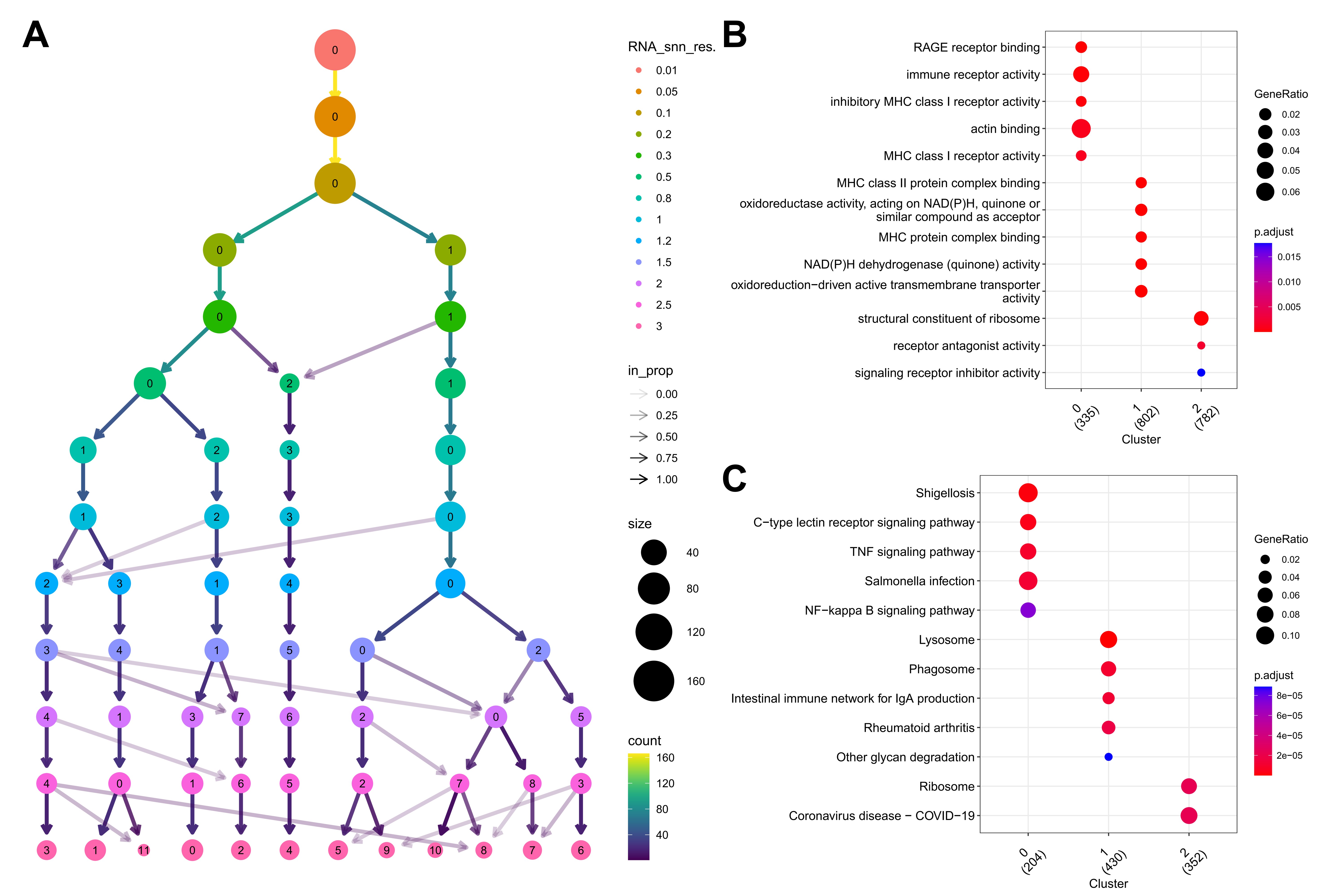

Supplement: Supplementary file 6 [file Image5.jpeg]

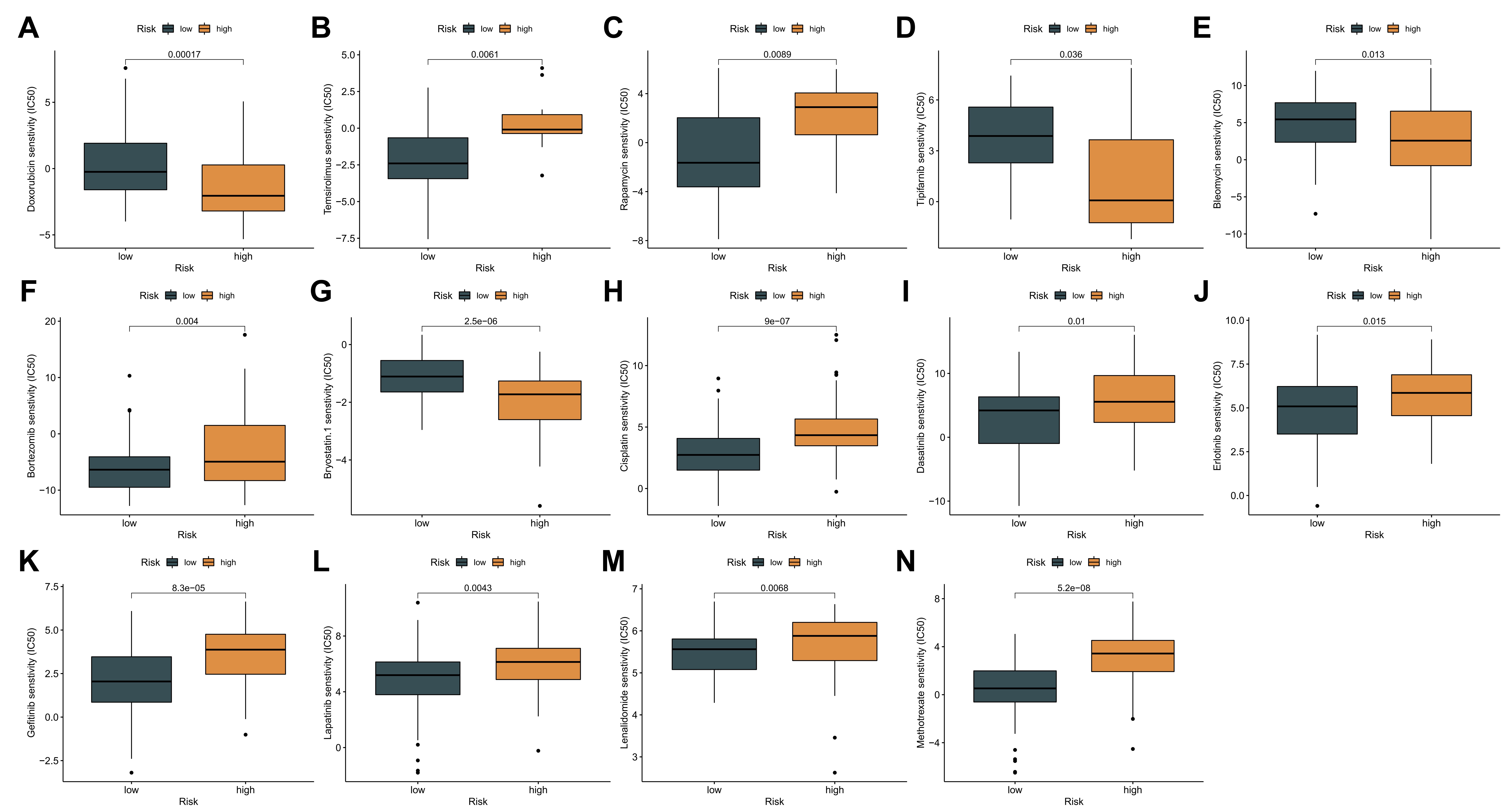

Supplement: Supplementary file 7 [file Image6.jpeg]

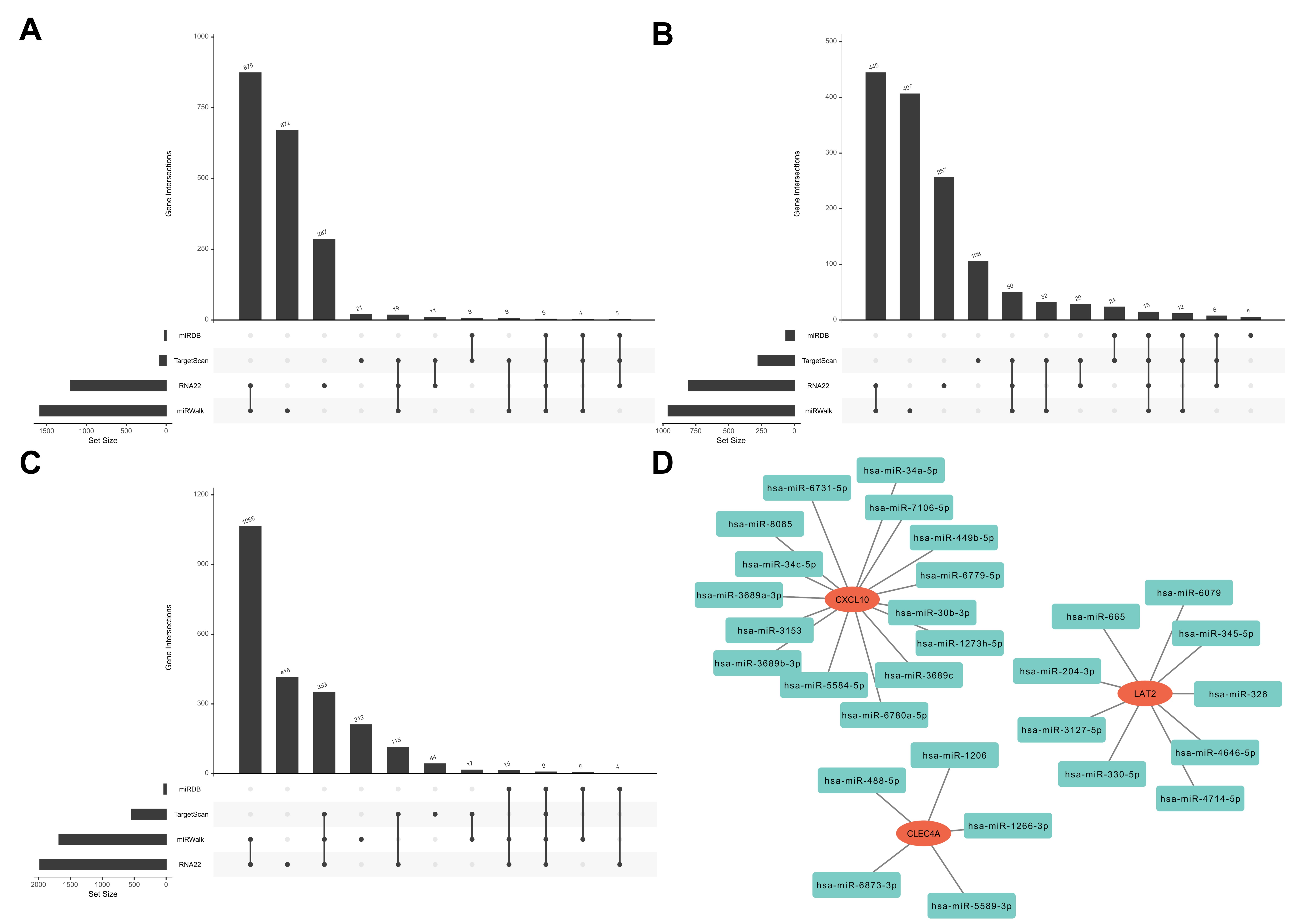

Supplement: Supplementary file 8 [file Image7.jpeg]

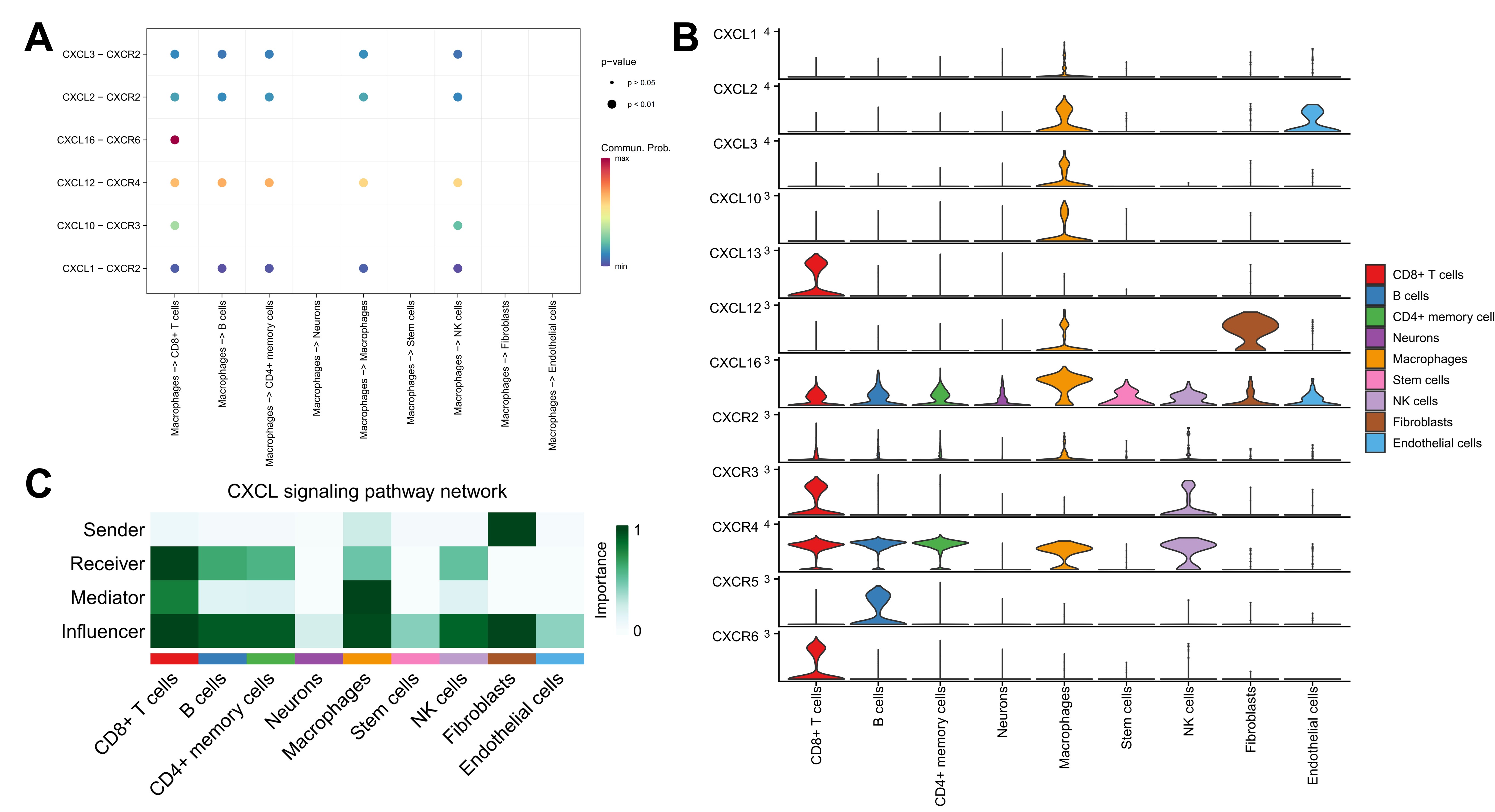

Supplement: Supplementary file 9 [file Image8.jpeg]
